# Emerging methods in biomarker identification for extracellular vesicle‐based liquid biopsy

**DOI:** 10.1002/jev2.12090

**Published:** 2021-05-12

**Authors:** Yaxuan Liang, Brandon M. Lehrich, Siyang Zheng, Mengrou Lu

**Affiliations:** ^1^ Center for Biological Science and Technology, Advanced Institute of Natural Sciences Beijing Normal University at Zhuhai Zhuhai China; ^2^ Medical Scientist Training Program University of Pittsburgh School of Medicine and Carnegie Mellon University Pittsburgh Pennsylvania USA; ^3^ Department Biomedical Engineering Carnegie Mellon University Pittsburgh Pennsylvania USA; ^4^ Department of Electrical and Computer Engineering Carnegie Mellon University Pittsburgh Pennsylvania USA

**Keywords:** biomarkers, biosensing, EV‐based diagnosis, exosomes, extracellular vesicles, liquid biopsy, microfluidics

## Abstract

Extracellular vesicles (EVs) are released by many cell types and distributed within various biofluids. EVs have a lipid membrane‐confined structure that allows for carrying unique molecular information originating from their parent cells. The species and quantity of EV cargo molecules, including nucleic acids, proteins, lipids, and metabolites, may vary largely owing to their parent cell types and the pathophysiologic status. Such heterogeneity in EV populations provides immense challenges to researchers, yet allows for the possibility to prognosticate the pathogenesis of a particular tissue from unique molecular signatures of dispersing EVs within biofluids. However, the inherent nature of EV's small size requires advanced methods for EV purification and evaluation from the complex biofluid. Recently, the interdisciplinary significance of EV research has attracted growing interests, and the EV analytical platforms for their diagnostic prospect have markedly progressed. This review summarizes the recent advances in these EV detection techniques and methods with the intention of translating an EV‐based liquid biopsy into clinical practice. This article aims to present an overview of current EV assessment techniques, with a focus on their progress and limitations, as well as an outlook on the clinical translation of an EV‐based liquid biopsy that may augment current paradigms for the diagnosis, prognosis, and monitoring the response to therapy in a variety of disease settings.

## 1 INTRODUCTION

**FIGURE 1:**
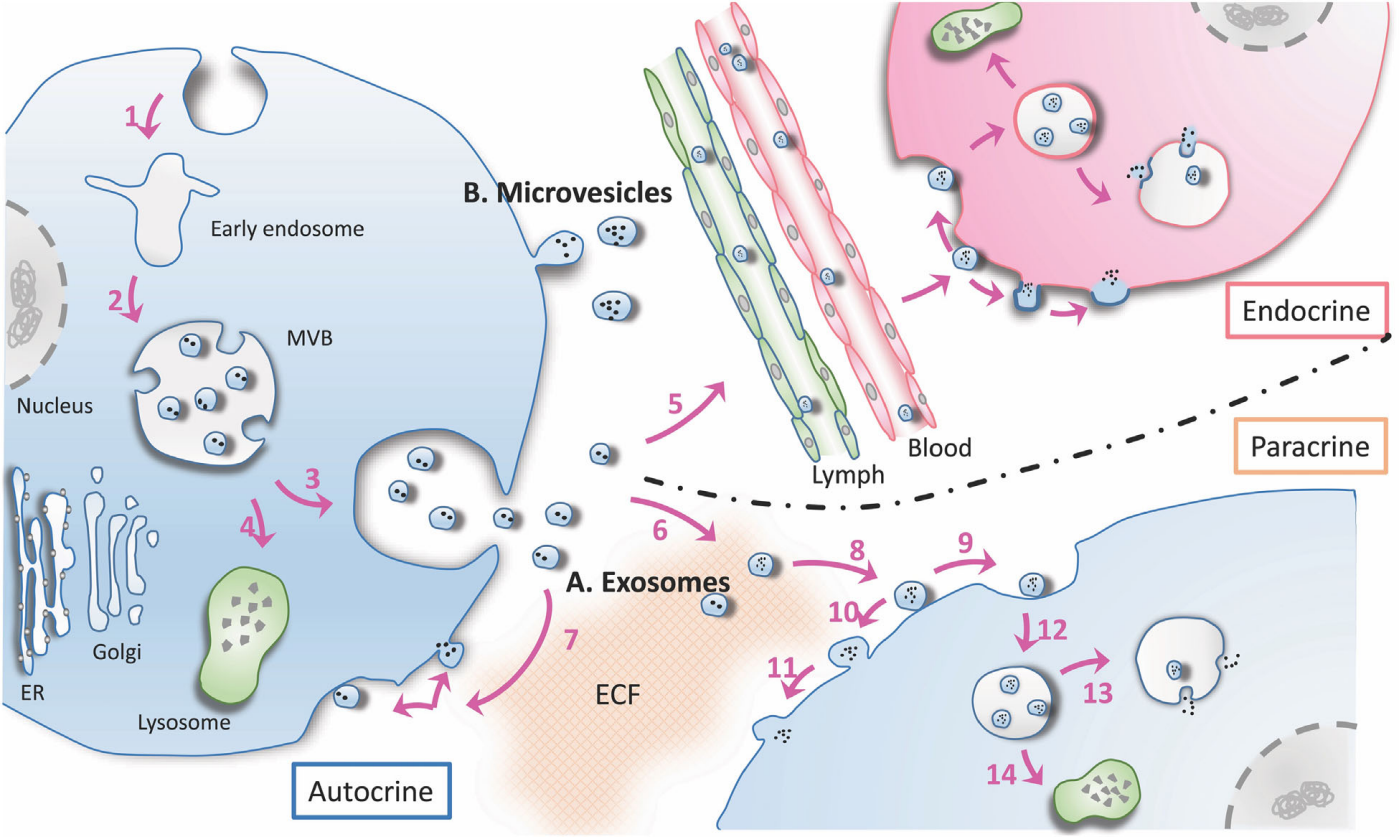
Schematic representation of EV biogenesis and cellular uptake. Though both considered as EVs, microvesicles and exosomes have distinct mechanisms of biogenesis. Exosomes (A) originate from the endosomal membrane system, where plasma membrane invagination forms the early endosome (1) and further maturates into multivesicular bodies (MVBs) (2). MVBs containing sacks of intraluminal vesicles (ILVs) either fuse to the plasma membrane to release exosomes (3), or are degraded and recycled by the lysosome (4). Secreted EVs can be taken up by cells of other tissues or distant organs via system circulation (endocrine) (5), by neighbouring cells through extracellular fluid transport (paracrine) (6), or by their cell of origin (autocrine) (7). Once EVs target and dock on the surface of recipient cells (8), they can undergo endocytosis (9), or directly fuse to the plasma membrane (10) and release their contents (11), depending on different mechanisms. Endocytosed EVs (12) release their contents through fusion within the endosomal membrane (13), but they may also be degraded by the lysosome via distinct signalling pathways (14). Microvesicles (B) are produced from the outward budding of plasma membrane constituents into the extracellular environment

Extracellular vesicles (EVs) are lipid bilayer membrane packets that are sustainably secreted from all eukaryotic cells and most known bacteria and archaea species (Deatherage & Cookson, 2012). In multicellular organisms where cellular crosstalk represents an essential element of physiology, EVs behave as versatile messengers mediating the conveyance of biological information from origin to recipient cells. Though thousands of various RNAs, proteins, and lipid species have been identified in the lumen or on the membrane of EVs (Kim et al., 2013; Kim et al., 2015), an explicit molecular catalog of an individual EV is still elusive due to vast heterogeneity within EV subpopulations. The complexity of EV subgroups is initiated from diverse parent cell types and their physiologically normal, activated, or stress states (Lasser et al., 2018; Willms et al., 2016). Significant quantities of miscellaneous EVs disperse in nearly all biofluid types, including blood, urine, saliva, cerebrospinal fluid, breast milk, and others. Within these complex biofluids, EVs traffic to target cells in bulk flow, transmit their cargo molecules, and subsequently alter the recipient cell's fate through endocrine, paracrine, or autocrine signalling (Figure 1). The assorted and abundant cargo molecules (i.e., nucleic acids, proteins, and lipids) associated with EVs originate from their elaborate endomembrane‐ or membrane‐assisted biogenesis pathways, based upon which researchers define three distinct subpopulations of EVs – termed exosomes, microvesicles, and apoptotic blebs (Jeppesen et al., 2019; Raposo & Stoorvogel, 2013). It is generally believed that exosomes are formed through inward invagination of endomembrane structures, forming intraluminal vesicles (ILVs) within multivesicular bodies (MVBs). The docking and fusion of the MVB to the cell's plasma membrane releases its ILVs into the extracellular fluid. Such vesicles are classified as exosomes (Figure 1A). On the other hand, microvesicles are thought to originate from direct outward extrusions or “buddings” from the plasma membrane with molecular cargos recruited from plasma membrane and cytosolic residues (Figure 1B). Lastly, the formation of apoptotic blebs occurs when cells are undergoing programmed cell death through distinct mechanisms explored elsewhere (D'Arcy, 2019). It should be noted that the heterogeneity of exosomes and microvesicles may exceed our general understanding as evidence has emerged that small exosomes (60–80 nm) and large exosomes (90–120 nm) may be distinct populations, and nanoparticles around 35 nm were found with a non‐membranous structure and as such termed exomeres (Zhang et al., 2018). Particularly, exomeres may be co‐pelleted with other vesicles via ultracentrifugation (Zhang et al., 2019). Therefore, to avoid the misinterpretation we tentatively define the family of EVs as consisting of all subgroups of exosomes and microvesicles throughout this review, although most of the reviewed articles do not distinguish between specific EV subpopulations with even the possibility of including a mixture of exomeres.

Circulating EVs in complex biofluids may serve as valuable disease biomarkers for diagnosis, prognosis, and monitoring response to therapy in various disease states. Recently, investigations have deemed such vesicles to have superior candidacy compared to free‐floating nucleic acids, proteins, and metabolites, as a noninvasive liquid‐biopsy for disease diagnosis, prognosis, and monitoring response to therapy (Hur et al., 2019; Wan et al., 2018). In this review we aim to focus on the recent methodological advancements made towards improving an EV‐based liquid biopsy. Improvements in EV detection, mRNA/miRNA profiling, and protein characterization have been increasingly reported throughout the literature and will be described here. Innovative techniques in studying EV lipid signatures have relatively been lacking in development, but we will discuss current improvements in mass spectrometry for EV lipidomics. Further, the significance of EV reference materials for EV translational prospects will be emphasized. Throughout this review we aim to illustrate the multidisciplinary efforts put forth towards improving the technological limitations of detecting vesicular entities, and highlight the journey towards EVs being utilized in clinical practice for a robust liquid biopsy.

## 2 EMERGING METHODS IN EV ASSESSMENT

EVs in complex biofluids are ideal candidates for a noninvasive liquid‐biopsy due to their easily accessible locations and their packaging of key molecular cargos. Conventional workflows to analyze EV biomarkers involve prior isolation of EVs from biological samples, and subsequent evaluation of EV‐associated nucleic acids, proteins, and/or lipids. However, the low recovery efficiency of current isolation methods for EV enrichment has restricted to a large extent the detection and quantification of lowly expressed EV molecules. Thus, innovative technologies with efficient enrichment of EVs, and sensitive cargo molecule detection are in great demand for their application in EV‐based diagnosis. Recently, various nanotechnologies and biosensing platforms have been developed and reported, providing opportunities to achieve low‐limits of detection and characterization of EV biotargets.

### 2.1 Determination of EV number concentration

Though not detecting specific EV‐associated molecules, counting the number of EVs per unit volume (defined as EV number concentration in this review) of a particular biological sample with comparison to a reference is still of clinical value according to recent evidence that the number concentration of EVs secreted into biofluids correlates well with increased disease severity and progression (Becker et al., 2016; Boriachek et al., 2017; Lane et al., 2018). Currently, commercial technologies to perform the characterization of EV number concentration include nanoparticle tracking analysis (NTA; e.g., NanoSight, ZetaView, or Horiba ViewSizer 3000) and resistive pulse sensing (RPS; including tunable RPS, e.g., Izon qNano Gold, and microfluidic RPS, e.g., Spectradyne nCS1). The measurement with these techniques commonly requires preparation of a purified EV sample before loading onto the instrument because their size‐dependent principle is not able to differentiate EV from non‐EV particles in the sample. Additionally, considering the moderate throughput (time consumed for measuring each sample), limited detecting range for nanoparticle number concentration (down to 10^6^–10^7^ particles/ml), and insufficient sensitivity to small EVs (with diameters < 50 nm), the above‐mentioned NTA and RPS techniques are not further detailed here. In the following subsections, we cover alternative options for the determination of EV number concentration that provides simplified sample preparation, improved measuring throughput, and/or enhanced detection sensitivity. Details of the below‐mentioned and other methods of determining the EV number concentration are summarized in Table 1.

**TABLE 1:** Summary of new techniques for the measurement of EV number concentration

Technique	EV source	EV isolation	EV capture	Probe	Sample volume	Detection instrument required	Ref.
Nanoscale Flow Cytometry	Rat plasma	None	None	Fluorescence dye	non‐specified	Customized cytometer	(Stoner et al., 2016)
Nanoscale Flow Cytometry	Cell culture, human plasma	UC	None	Fluorescence Ab	non‐specified	Lab‐built instrument	(Tian et al., 2018)
Nanoscale Flow Cytometry	Cell culture	SEC or UC	None	Fluorescence dye or Ab	non‐specified	CytoFLEX system, Beckman Coulter	(Choi et al., 2019)
Nanoscale Flow Cytometry	Cell culture	UC+SEC	None	Fluorescence dye	non‐specified	Astrios EQ, Beckman Coulter	(Morales‐Kastresana et al., 2017)
Redox cycling + enzymatic reaction to amplify electrochemical signals	Cell culture, platelet concentrate	Supernatant from 2000 x *g*	EpCAM‐Ab functionalized platinum electrode	EpCAM‐Ab with alkaline phasphotase	As low as 25 μl EV suspension	Fabricated electrodes	(Mathew et al., 2020)
Rolling circular amplification + hemin/G‐quadruplex mediated electrochemical detection	Human plasma	UC	CD63‐Ab functionalized gold electrode	Aptamer‐primer	non‐specified	Fabricated electrodes	(Huang et al., 2019)
Novel probe + SPR	Cell culture	UC	CD63‐aptamer modified gold film	Dual AuNP with electronic coupling	non‐specified	EC‐SPR device, Dingcheng Technology	(Wang et al., 2019)
Novel probe + SERS	Human plasma	UC	EpCAM‐aptamer modified magnetic MB	AuNP in triangular pyramid DNA	1 μl of labelled EV suspension	Unspecified Raman microscope	(Zhang et al., 2019)
DNA hybridization chain reaction + TIRF imaging for single EV counting	Mice/human plasma	None	CD63‐Ab functionalized glass slides	Aptamer‐based DNA nano‐device	1 μl plasma diluted in 10 μl buffer	Lab‐built TIRF microscope with commercial components	(He et al., 2019)
Interferometric imaging + digital counting and sizing single vesicles	Cell culture, human CSF	Sucrose gradient UC	Ab functionalized silicon chips	None	20 μl EV sample	NVDX10 reader, Nexgenarrays LLC	(Daaboul et al., 2016)
Fluorescence polarization assay	Human plasma	None	None	Dye‐labelled aptamer	Less than 1 ul plasma diluted in 50 μl buffer	Infinite M1000 PRO plate reader, Tecan	(Zhang et al., 2019)
CTSDR‐based DNA catalytic reaction + electrochemical detection	Cell culture	ExoEasy Maxi Kit, Qiagen	CD63‐Ab modified MB	CD63‐aptamer	25 μl raw EV isolates	Fabricated electrodes	(Cao et al., 2019)
HRP‐induced fluorescence + photonic crystal‐assisted sensing	Human serum	Nanoporous double filtration	None	CD63‐aptamer	20 μl serum	QE 65000 fiber optic spectrometer, Ocean Optics	(Dong et al., 2019)
Reduced graphene oxide field effect transistor biosensor + electric signal detection	Human serum	UC	CD63‐Ab modified graphene substrate	None	10 μl	Probe station, EverBeing BD‐6 and Keithley 4200‐SCS	(Yu et al., 2019)

Abbreviations: Ab, antibody; AuNP, gold nanoparticle; CSF, cerebrospinal fluid; CTSDR, cascade toehold‐mediated strand displacement reaction; MB, magnetic beads; SEC, Size exclusion column; SERS, Surface‐enhanced Raman spectroscopy; SPR, surface plasmon resonance; TIRF, total internal reflection fluorescence microscopy; UC, ultracentrifugation.

#### 2.1.1 Nanoscale flow cytometry of EVs

Conventional flow cytometry has been practiced in clinical laboratories for patient care. For characterizing EVs with small sizes, most ordinary cytometry instruments will need an upgrade with optimized configurations to meet the detection sensitivity. The challenge with flow cytometry of EVs arises from i) the small particle sizes and ii) the low refractive index, which significantly diminish the light scattering intensity, and thus leave the triggering threshold difficult to set against background signals. One way to address this issue is to employ fluorescence as a trigger parameter, which offers advantages in lowering noise signals from the cytometry instrument and buffers (Arraud et al., 2016; Nolan, 2015; Stoner et al., 2016). However, it requires additional steps for EV fluorescent labelling. Other groups aim their attention at customizing commercial cytometry instruments (Groot Kormelink et al., 2016; Higginbotham et al., 2016; van der Vlist et al., 2012) or completely building a homemade cytometry instrument (Tian et al., 2018) with intention to improve the laser power and the detector sensitivity (e.g., addition of a high‐performance photo multiplier tubes [PMT] for collecting the forward scatter). More elaborate hardware designs further consider narrowing down the sample stream flowing and extending the holding time of a vesicle in the laser beam to accumulate photon collection (Tian et al., 2018). Another reported technique recruited in EV detection is imaging flow cytometry, which has PMT replaced by a charge coupled device (CCD) to avoid requirement of trigging and further lowers the background noise with image confirmation (Lannigan & Erdbruegger, 2017). Collectively, compared to most other EV analytic methods, flow cytometry of EVs has advantages in analysis throughput (up to ten thousands of events per second). Additionally, some reported high‐resolution flow cytometry is able to detect EVs with a diameter down to 40 nm (Tian et al., 2018). Multiplex immunolabeling of EV cargo molecules (e.g., proteins, nucleic acids, etc.) accompanied with sizing and refractive index features further allows the flow cytometry to subtype EV populations in multiple dimensions (Choi et al., 2019; Morales‐Kastresana et al., 2017). Nonetheless, flow cytometry of EVs for number concentration is subject to a few issues. For instance, EV purification is commonly required before flow analysis and thus variations in the recovery and purity of EV isolates across batches and operators result in arduous recapitulation of the actual EV number concentration from clinical samples. Fluorescent labelling and washing further results in unknown sample loss. Additionally, despite enhanced sensitivity, most nanoscale cytometry instruments do not give reliable measurements to EVs smaller than 50 nm. Taking these considerations, nano‐flow researchers may be encouraged to consider well‐designed spike‐in controls and to cross‐reference with other methods to further optimize a prevalent methodology for the measurement of EV number concentration when moving towards clinical translation.

#### 2.1.2 Biosensor for signal transduction and amplification

**FIGURE 2:**
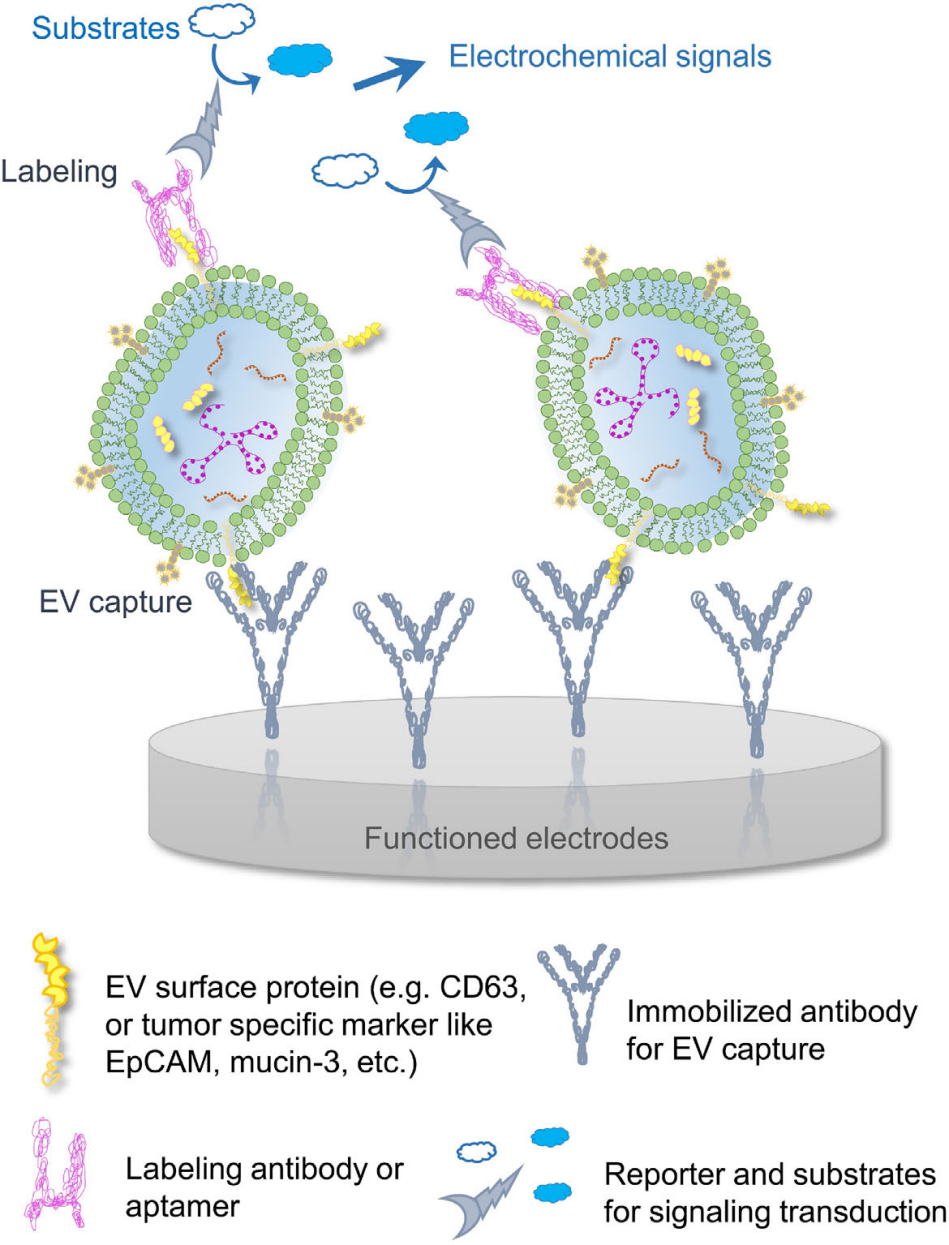
Schematic of EV concentration biosensing platform featured for “Signal Transduction and Amplification”. The technique typically includes the steps of EV capture, reporter labelling, and electrochemical signals transducing

Advanced biosensing platforms have emerged to provide EV measurement with ameliorated limit of detection (LOD) for EV number concentration. The strategy most reported biosensors employ is based on signal transduction and amplification, with typical steps of capturing EVs, labelling signal‐transducing agents, and recording electric/optical output (Figure 2). Mathew et al. reported a sophisticated sensing scheme that captured and immobilized tumour‐derived EpCAM^+^ EVs on electrodes followed by immunolabeling of alkaline phosphatase on the EV surface. The phosphatase cleaves the substrate to generate electrochemically active entities, which then convert and amplify the signal to a steady‐state current (Mathew et al., 2020). This sensing assay was reported to detect the number concentration of tumour EVs as low as 5×10^3^ EVs/ml within a linear range of 1×10^4^ to 1×10^9^ EVs/ml (Mathew et al., 2020). Another EV biosensor design that premised on a similar idea captured and immobilized CD63^+^ EVs, and identified gastric cancer cell derived EVs by an aptamer probe containing a mucin‐3 binding region. The EV‐bound probe then triggered a rolling circle amplification (RCA), a novel nucleic acid amplification technology that generated a long DNA single strand that can fold to form a large quantity of G‐quadruplex units. Upon incubation with hemin and H_2_O_2_ substrates the system produced an electrochemical signal proportional to the EV number concentration in the original sample, leading to a sensitive biosensing platform for the quantification of EV number concentration (Huang et al., 2019). This biosensor design was estimated to detect mucin‐3^+^ EVs as low as of 9.54 × 10^2^ EVs/ml, and in a linear range of 4.8 × 10^3^ to 4.8 × 10^6^ EVs/ml (Huang et al., 2019). Taken together, these sensing designs utilize signal transduction and electrochemical amplification to determine the EV number concentration. The integration of EV capture with analysis theoretically does not require a pre‐purification step, however, many current schemes prefer to apply pure EV samples instead of being well prepared for a raw and complex clinical specimen. Nevertheless, most designs demonstrate a robust performance in terms of sensitivity of detecting low‐number EVs without resolution issues on particle size. This feature allows EV biosensing techniques as a good candidate for seizing scarce pathological EVs from bulk biofluids.

#### 2.1.3 SPR and SERS employment for EV detection

**FIGURE 3:**
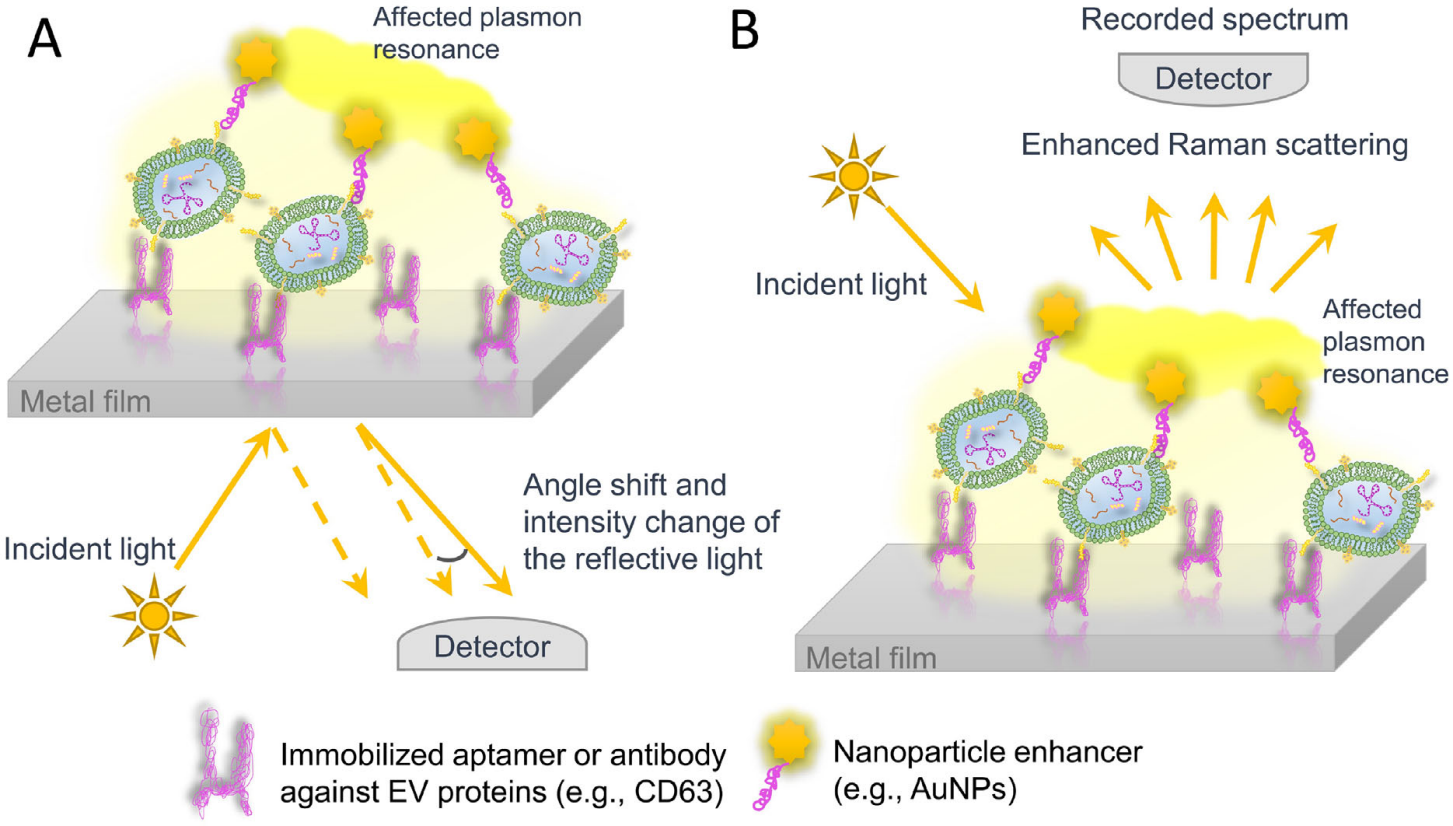
Techniques of SPR and SERS applied for the measurement of EV number concentration. (A) Representation for the principle of SPR technique. (B) Representation for the principle of SERS technique

Surface plasmon resonance (SPR) has been reported for the application of determining the EV number concentration (Im et al., 2017; Picciolini et al., 2018; Sina et al., 2019; Wang et al., 2019; Yang et al., 2020). The principles of the SPR technique rely on the excitation of oscillating free electrons occurring at the interface of a metal film and the medium (i.e., analytes) using a polarized light source. The angle shift of the reflective light caused by different analyte refractive indices is recorded subsequently for quantitation (Figure 3A). A reported application of SPR in measuring the EV number concentration utilized a CD63‐binding aptamer functionalized Au film for EV capture, and then the immobilized EV was further labelled with Au nanoparticles (AuNP) via hybridization of complimentary oligonucleotides. The abundance of captured EVs on the Au film surface results in a specific reflective light intensity and angle shift, and the decoration of AuNPs on the surface of EVs enhances plasmonic effects, together making a sensitive detection of EV number concentration down to 5 × 10^3^ particles/ml (Wang et al., 2019).

Interestingly, similar approaches that utilize surface‐enhanced Raman spectroscopy (SERS) have been reported to enhance and complement EV detection by several groups (Carmicheal et al., 2019; Kwizera et al., 2018; Shin et al., 2018; Stremersch et al., 2016; Zhang et al., 2019). SERS is an augmented Raman Spectroscopy technique developed for molecules with low concentrations (e.g., down to picomolar scale). This technique takes advantage of resonant oscillation of conducting electrons (i.e., plasmon resonance) on certain metal surfaces stimulated by incident light. Signals from SERS are several orders of magnitude higher than normal Raman scattering. Depending on the light absorbance property of each individual molecule, the spectrum of scattered photons will be recorded for analyte identification (Figure 3B). In the study reported by Zhang et al., a novel Raman probe was developed to enhance the signal from circulating tumour EVs of low number concentrations. Specifically, EVs were initially enriched on magnetic beads (MB) that were modified with aptamers against EpCAM, which is a cancerous marker known to be prevalent on the surface of tumour‐derived EVs. The MB‐bound EVs were further labelled with a strengthened Raman probe which had a DNA‐assembled triangular pyramid nanostructure with AuNPs clustered in DNA tetrahedrons (Zhang et al., 2019). Assisted by SERS technique and the improved probe, the detection of circulating cancerous EVs reached down to 1.1 × 10^5^ particles/ml as well as providing a high selectivity for EpCAM^+^ EVs (i.e., separating breast cancer patients from healthy individuals). Ideally the SPR and SERS application for determining the EV number concentration is able to directly analyze the complex clinical samples with instruments potentially standardized and commercialized, which would be positive for future clinical practice.

#### 2.1.4 Direct counting of single vesicles

**FIGURE 4:**
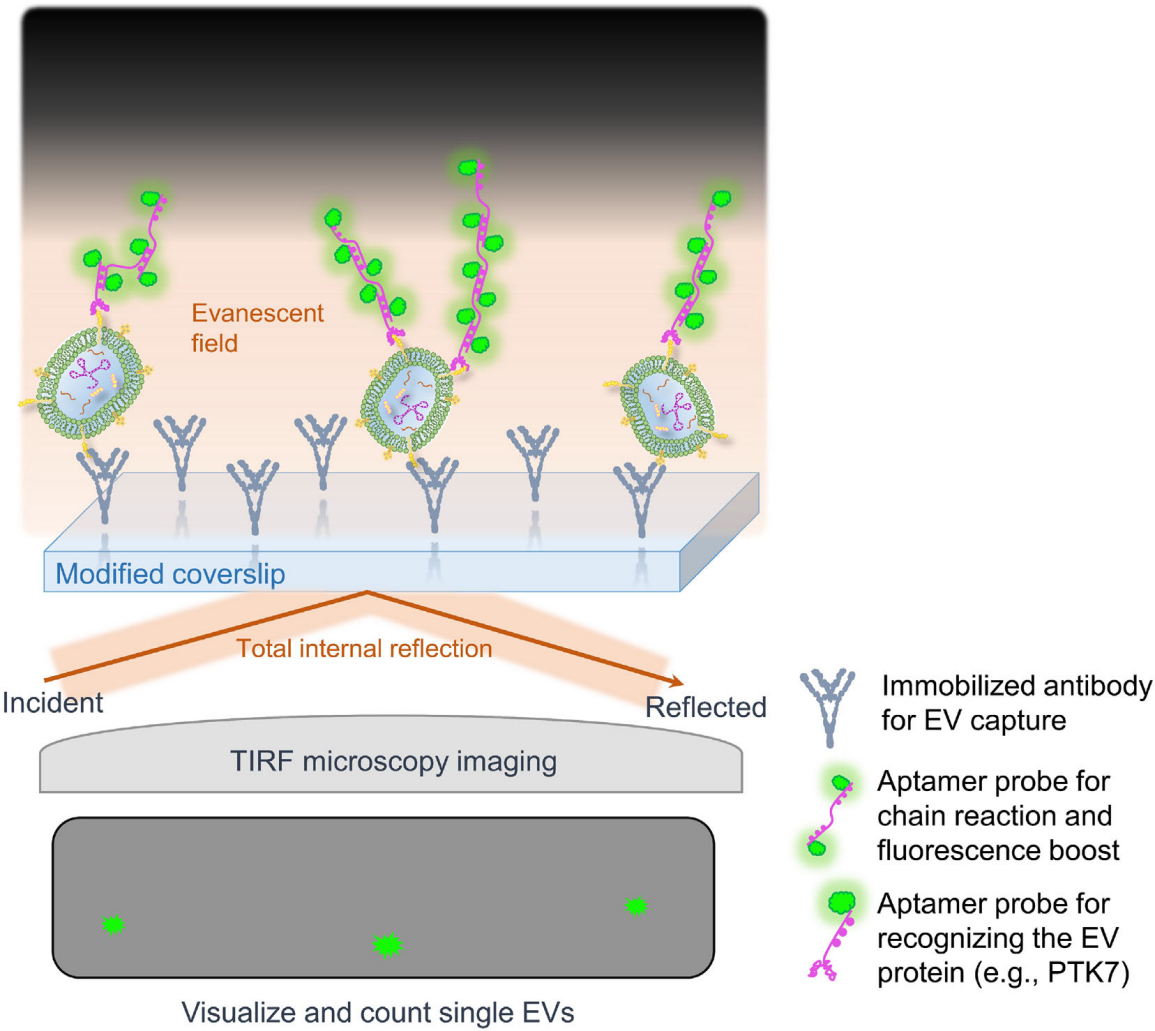
An example of single vesicle visualizing and counting through amplified fluorescence. EVs are captured on a coverslip and labelled with fluorescence probes. DNA hybridization chain reaction with addition of substrates boosts fluorescence signals from each single vesicles, allowing for direct counting of EV number through TIRF microscopy

Visualizing and direct counting the number of single EVs provide another solution for determining the EV number concentration. Super‐resolution microscopy now is able to separate focal spots smaller than 100 nm (Pujals et al., 2019), while due to the complexity of the instrument and expertise required for the operator it was not considered as an optimal tool directly for the measurement of EV number concentration. NTA is another example of optical configuration for EV visualization and counting that takes advantage of scattering light, however, reliable measurement of dim vesicles with low refractive index and small vesicles with diameter lower than 60–70 nm is still under debate (Bachurski et al., 2019; van der Pol et al., 2014). To explore the possibility of imaging small EVs with more reliability, in situ magnification of fluorescent signals from a single vesicle as a potential solution was reported. He et al. attempted to exploit DNA hybridization chain reaction (HCR) to boost the fluorescence signal. The design included EV capture onto a CD63‐antibody modified coverslip, followed by HCR labelling. The labelling started with binding of an aptamer probe which consisted of three sequence domains: 1) a PTK7‐targeted aptamer sequence that recognized PTK7‐positve EVs (tumour origin), 2) a poly‐T linker, and 3) a DNA trigger for initiating a HCR event. In this specific reaction, two species of fluorophore‐labelled DNA hairpins alternatively open and bind to the stem strand, thus the intensely labelled fluorophores significantly amplify the readout of the fluorescent signals in situ (Figure 4). Finally, individual EVs were visualized and quantified by total internal reflection fluorescence (TIRF) microscopy, which can generate a shallow excitation (evanescent) field (e.g., 100–300 nm, depending on the settings) close to the interface of the coverslip and adherent EVs, while leaving the bulk solution silent (Figure 4) (He et al., 2019). TIRF microscopy combined with HCR labelling of EVs can generate single‐EV images with high signal‐to‐background ratio and allow detection of tumour EVs directly from the diluted plasma collected from tumour‐transplanted mice (He et al., 2019).

Other than fluorescence‐based EV visualization, researchers have explored the interferometric reflectance imaging method for single EV number counting. In the assay EVs were first captured by an antibody microarray immobilized on a silicon chip, followed by acquisition of interferometric images by a chip reader. Each single EV then appeared as a diffraction limited spot with brightness and contrast correlated to the size of the vesicle. Together with digital counting of diffraction spots, the statistics of EV number and sizes were able to be documented (Daaboul et al., 2016). Intriguingly, the interferometry instrument and sensor chips for EV characterization have been commercially manufactured (ExoView by NanoView Biosciences) with a range of EV‐associated antibodies available to capture specific subpopulations of EVs. According to the service provider, the assay is performed directly with the specimen without EV purification and is able to comprehensively characterize the EV number concentration, the EV size (within 50–200 nm range), and the EV protein expression, regardless of the potentially appreciable cost for the order of each assay.

#### 2.1.5 Limitations

Now various advanced nanotechnologies are being applied for characterizing EV number concentration with enhanced applicability and sensitivity to detect EV subpopulations. However, limitations with these technologies still exist. First, many of the reported EV number concentrations are dependent on EV size/density properties or specificity to particular EV markers (e.g., CD63), while it is still not well confirmed that all distinct EV subpopulations are within a certain size range, or positive for single protein markers. In fact, there may not exist a method to reveal the “true” number of EVs within a volume of biosample. NTA, RPS, and nano flow cytometry are designed for sensing EV subgroups within certain size ranges, while other methods may dedicate to catch EV subgroups bearing certain protein markers. From a broader perspective, this issue may be more associated with the consensus of an EV definition and a better understanding of EV biology within the community. Currently, we are still in demand for improved benchmarking methods to measure the EV number concentration in a biofluid with a known uncertainty. Second, the binding efficiency should be considered in the capture‐dependent methods when estimating the EV number concentration in reference to original samples. Spike‐in EV reference materials may be encouraged to minimize the variation resulting from EV enrichment steps. Third, as “proof‐of‐concept” attempts, most studies have shown less confidence to evaluate non‐pretreated clinical samples, particularly human plasma, which is one of the most complex biofluid sources of EVs. This has impeded to a certain degree their potential translational significance. Nonetheless, the integration of these cutting‐edge nanotechnologies are actively and rapidly opening new doors to further expand the potential for EV‐based liquid biopsy clinical translation.

### 2.2 EV RNA characterization

EVs are natural carriers for a variety of nucleic acids involving diverse RNA species. Many thousands of mRNAs, miRNAs, lncRNAs, tRNAs, rRNAs, and circular RNAs with different sequences have been documented in previous studies (Keerthikumar et al., 2016; O'Brien et al., 2020). Compared to a limited number of tools analyzing trace amounts of EV proteins or structurally diversified lipids, polymerase chain reaction (PCR)‐based amplification of nucleic acid copies allows for a higher probability to uncover EV RNA species with low abundances. Existing RNA analytical platforms have included reverse‐transcription quantitative PCR (RT‐qPCR), microarray, and next‐generation sequencing (NGS), while the assay workflow either requires multiple steps of sample processing, or complex library preparation and high sequencing depth. Nonetheless, the LOD is still difficult to approach the level of detecting certain sporadic RNAs in specific EV subpopulations, let alone to single nucleotide variants. Here, we attempt to review emerging EV RNA analytical platforms that either integrate and simplify processing steps, enhance the sensitivity to RNA molecules, or highlight specific RNA‐enriched EV subtypes. Information from these EV RNA technologies is summarized in Table 2.

**TABLE 2:** Summary of new techniques for EV RNA characterization

Technique	EV source	EV enrichment	RNA extraction	Target	Sensitivity	Detection instrument required	Ref.
Hybridization probe + digital PCR‐free assay	Cell culture	UC	RNAeasy kit, Qiagen	mRNA (GAPDH, EWS‐FLI1)	20 aM	Fluorescence microscope	(Zhang et al., 2018)
Multi‐colour fluorescence digital PCR EV‐lncRNA (miDER) analysis	Plasma bearing lung cancer	None	exoRNeasy Serum/Plasma Midi Kit, Qiagen	lncRNA (SLC9A3‐AS1, PCAT6)	10 copies/μl	Thermal cycler and fluorescence microscope	(Bai et al., 2019)
BEAMing RT‐PCR + digital PCR	Serum, CSF	UC	miRNeasy kit, Qiagen	mRNA (IDH1 mutant transcript)	non‐specified	Digital PCR system, RainDance Technologies	(Chen et al., 2013)
Localized gold nanoprisms + SPR	Cell culture, plasma	UC	TRIzol kit, Direct‐zol RNA MiniPrep kit	miRNA (miR‐10b)	aM with single nucleotide specificity	Varian Cary® 50 UV‐Vis Spectrophotometer, Agilent	(Joshi et al., 2015)
Head‐flocked gold nanopillar + locked nucleic acid probe + SERS	Cell culture	qEV size exclusion column, Izon	Total Exosome RNA and Protein Isolation Kit, Invitrogen	miRNA (miR‐21, miR‐222, miR‐200c)	aM with single nucleotide specificity	Customized Raman microscopy, NOST, Gyeonggi‐do	(Lee et al., 2019)
Duplex‐specific nuclease mediated signal amplification + SERS	Plasma	Total exosome isolation, Reagent, Life Technologies	TRIzol RNA kit, Life Technologies	miRNA (miR‐10b)	aM with single nucleotide specificity	Portable Raman spectrometer, B&W Tek	(Pang et al., 2019)
Microfluidic chip integrating surface acoustic wave‐based EV lysis and RNA sensing	Cell culture	None	None	miRNA (miR‐550)	2 pM	Lab‐fabricated device	(Taller et al., 2015)
Microfluidic chip integrating immunomagnetic selection, RNA collection and real‐time PCR	Serum	CD63‐, EGFR‐Ab MB	Glass bead filter capture	mRNA (MGMT, APNG)	Regular qPCR level	Lab‐fabricated microfluidic device	(Shao et al., 2015)
DNAzyme probe penetration + TIRF	Cell culture, serum	ExoQuick™ kit, SBI	None	miRNA (miR‐21, miR‐221)	Single vesicle and stoichiometric miRNA number	prism‐type TIRF microscope, lab‐built with commercial components	(He et al., 2019)
Catalyzed hairpin DNA circuit probe in cationic lipid‐polymer hybrid nanoparticles + TIRF	Cell lines, serum	UC for cell cultures, None for serum; captured by electrostatic, Interactions	None	mRNA (glypican‐1)	Quantified by arbitrary unit	TIRF microscope, Nikon Eclipse Ti Inverted Microscope System	(Hu et al., 2017)
Virus‐mimicking fusogenic vesicle‐mediated molecular beacon + Flow cytometry	MCF7 cell culture, serum bearing breast cancer	UC	None	miRNA (miR‐21)	Single vesicle subtyping	Flow cytometer, LSR Fortessa analyzer, BD Biosciences	(Gao et al., 2019)
Light‐driven self‐powered device	Serum	ExoQuick™ kit, SBI	miRNeasy Micro Kit, Qiagen	mRNA (HOTTIP)	5 fg/ml	Fabricated electrodes	(Pang et al., 2019)

Abbreviations: Ab: antibody; MB: magnetic beads; SBI: System Biosciences Inc.; SERS: Surface‐enhanced Raman spectroscopy; SPR: surface plasmon resonance; UC: ultracentrifugation. Units: 1 aM = 1 × 10^–18^ M, 1 pM = 1 × 10^–12^ M, 1 fg/ml = 1 × 10^–15^ g/ml.

#### 2.2.1 Digital detection of single RNA molecules

**FIGURE 5:**
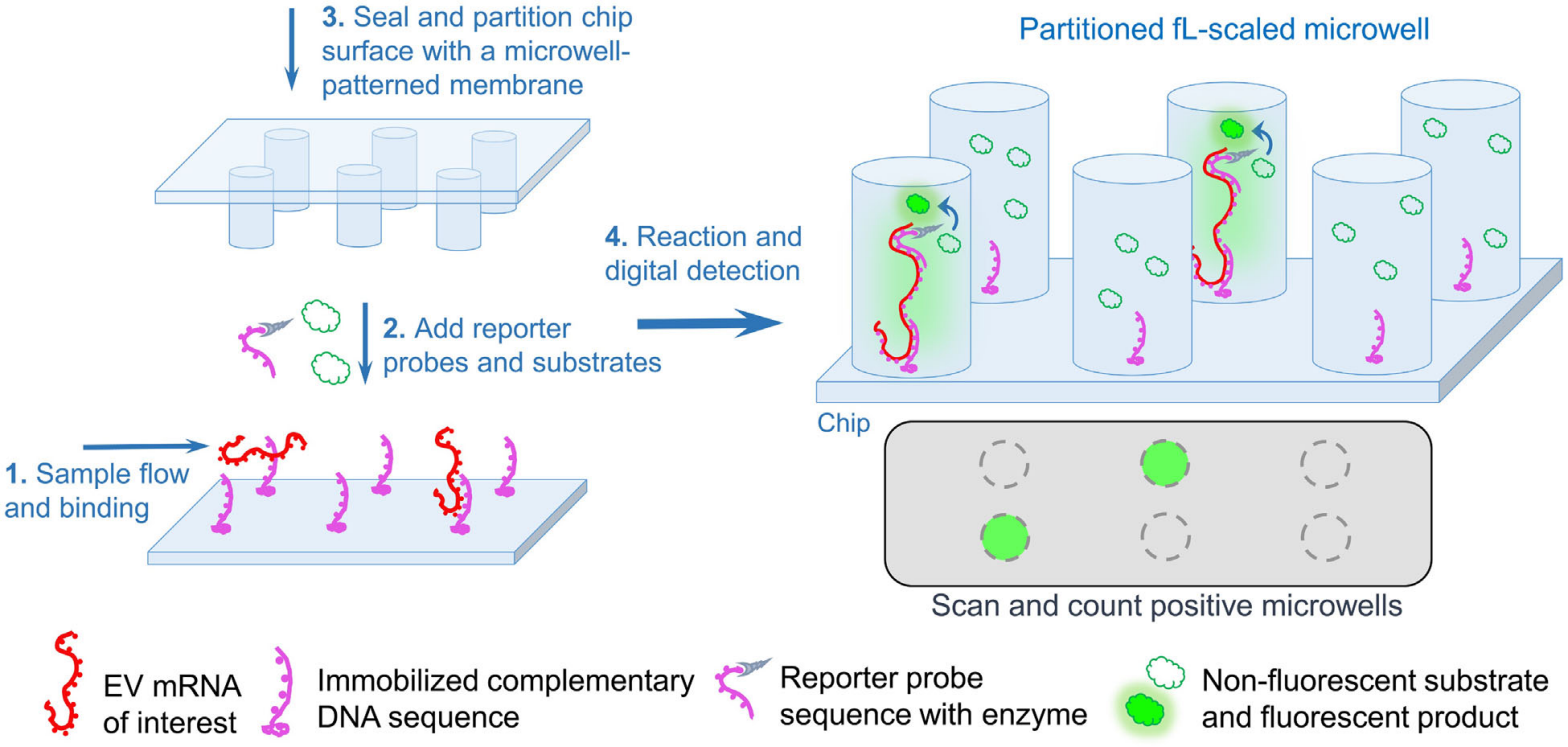
A PCR‐free digital assay for EV mRNA detection. EV RNAs flow through a complementary DNA‐modified chip (1), where EV mRNAs of interest bind and stick on the chip. Following addition of reporter probes and substrates (2), the chip is sealed and partitioned into fL‐scaled microwells (3). Detectable fluorescence signals are generated within EV mRNA‐positive microwells, allowing for digital reading copy numbers of target mRNAs (4)

Digital bioassays are designed to partition the sample mix into a large number of discrete small compartments, normally from the femtoliter (10^–15^; fL) to nanoliter (10^–9^; nL) scale in volume, with most individual compartments containing zero or one target molecules in accordance with Poisson statistics. After specific amplification reactions (e.g., PCR, HCR, RCA, or others), signals from target‐positive compartments are then detectable. By counting quantities of target‐positive and target‐negative compartments, absolute quantification of target molecules of interest in the analyte can be achieved through Poisson statistics. Digital bioassays offer superior sensitivity and accuracy without the need for a reference standard and/or endogenous control. In a recently reported study, EV mRNAs were digitally examined on a PCR‐free microfluidic platform which integrated target capture, probe tagging, and fluorescence signal production (Zhang et al., 2018). Specifically, the extracted EV RNA mix flowed through the chip where the mRNAs of interest were captured by hybridizing with immobilized DNA probes. Then, the reporter probes bound and labelled the targets with β‐galactosidase (fluorogenic enzyme) and fluorescein di‐β‐D‐galactopyranoside (non‐fluorescent substrate), followed by immediately sealing and partitioning the chip surface into a large number of confined volumes via a microwell‐patterned membrane. In individual fL‐scaled microwells, the enzymatic fluorogenic reaction generated detectable signals for later digital counting (Figure 5). The method reported 64.6–43.5 copies of GAPDH mRNA and 6.5–0.277 copies of EWS‐FLI1 fusion transcripts per 10^5^ EVs (Zhang et al., 2018).

Among digital assays, digital PCR (dPCR) is one of the most widely‐used technologies, and a few dPCR systems have been commercially available for DNA/RNA absolute quantification. Meanwhile, an increasing number of laboratories has been attracted towards utilizing dPCR systems for EV RNA study. Profiling EV RNA via dPCR systems from biofluids have been demonstrated to predict treatment response and disease progression. Takahashi et al. described and optimized the protocol for miRNA and lncRNA dPCR analysis for EVs derived from cell culture medium and serum (Ferracin & Negrini, 2018; Takahashi et al., 2014), while other groups have carefully compared the performance between dPCR and qPCR for EV miRNA detection in the setting of chronic diseases and cancers, and concluded that dPCR outperformed qPCR in terms of LOD (i.e., quantification of RNA copy numbers), consistency, and reproducibility (Bellingham et al., 2017; Wang et al., 2019). However, existing commercial dPCR systems are not without their flaws, including high‐cost instrumentation, limitations in multiplexing capability, and low throughput. Bai et al. attempted to address some of these issues by developing an on‐chip dPCR platform for EV lncRNA analysis. Intriguingly, the system employed multiplex PCR techniques on a homemade microfluidic chip, enabling simultaneous detection of several target EV lncRNAs in a single run (Bai et al., 2019). Another challenge associated with current dPCR systems is the detection of rare single nucleotide mutants among dominant wild‐type sequences from clinical EV samples. Recently Chen et al. reported a novel strategy combining BEAMing (beads, emulsion, amplification, magnetics) PCR and dPCR for identification of EV‐carried mRNA transcript mutants. In this assay, researchers robustly detected *IDH1* mRNA mutants from cerebrospinal fluid‐derived EVs that were collected from patients bearing glioma tumours, manifesting the efficiency of the enhanced dPCR system (Chen et al., 2013).

#### 2.2.2 Application of SPR and SERS techniques

**FIGURE 6:**
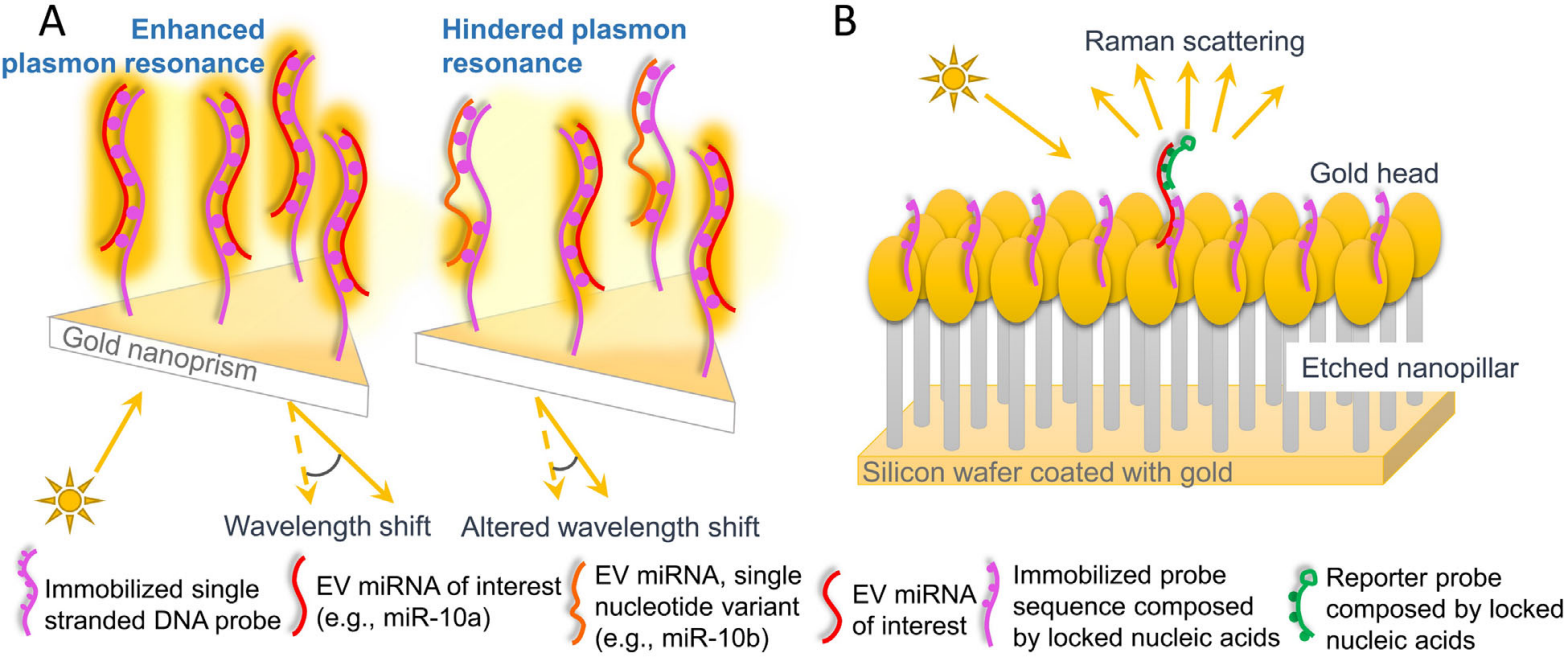
Technique schematics for EV RNA characterization utilizing SPR and SERS. (A) An example of SPR nanobiosensor applied for EV miRNA measurement. A gold nanoprism‐structured SPR sensor is fabricated to capture the EV miRNA of interest. Upon hybridization, the double helix structure alters the detected SPR with single‐nucleotide specificity. (B) SERS application for EV miRNA measurement. A nanopillar‐structured substrate is functionalized with locked nucleic acids on the Au head for EV miRNA capture. Then, the miRNA of interest is labelled with a probe that augments the SERS detection

Attributed to the sensitive signal transducing, SPR and SERS techniques have been adapted towards EV RNA evaluation. In fact, SPR based RNA biosensors have been developed with a variety of sophisticated plasmonic probes and signal transduction techniques (Aoki et al., 2019; Coutinho & Somoza, 2019; Fong & Yung, 2013; Xue et al., 2019), and such methods were applied for analyzing assorted biological samples, for instance, urinary miRNA (Yeung et al., 2018) and Zika viral RNA (Adegoke et al., 2017). However, few published investigations have focused on EV RNA characterization. Joshi et al. recently reported a gold nanoprism‐assisted SPR biosensor for EV miRNA detection. Upon hybridizing with immobilized complementary probes, the newly formed double helix structure increased the local refractive index near the gold nanoprism, resulting in a wavelength shift (Figure 6A). Intriguingly, the design was able to differentiate miR‐10b from miR‐10a according to the principle that the site with unbound base pairs hindered electron transport, thus shifting the SPR pattern (Joshi et al., 2015). The reported SPR biosensor managed to detect higher miR‐10b expression in plasma EVs collected from pancreatic cancer patients (Joshi et al., 2015).

**FIGURE 7:**
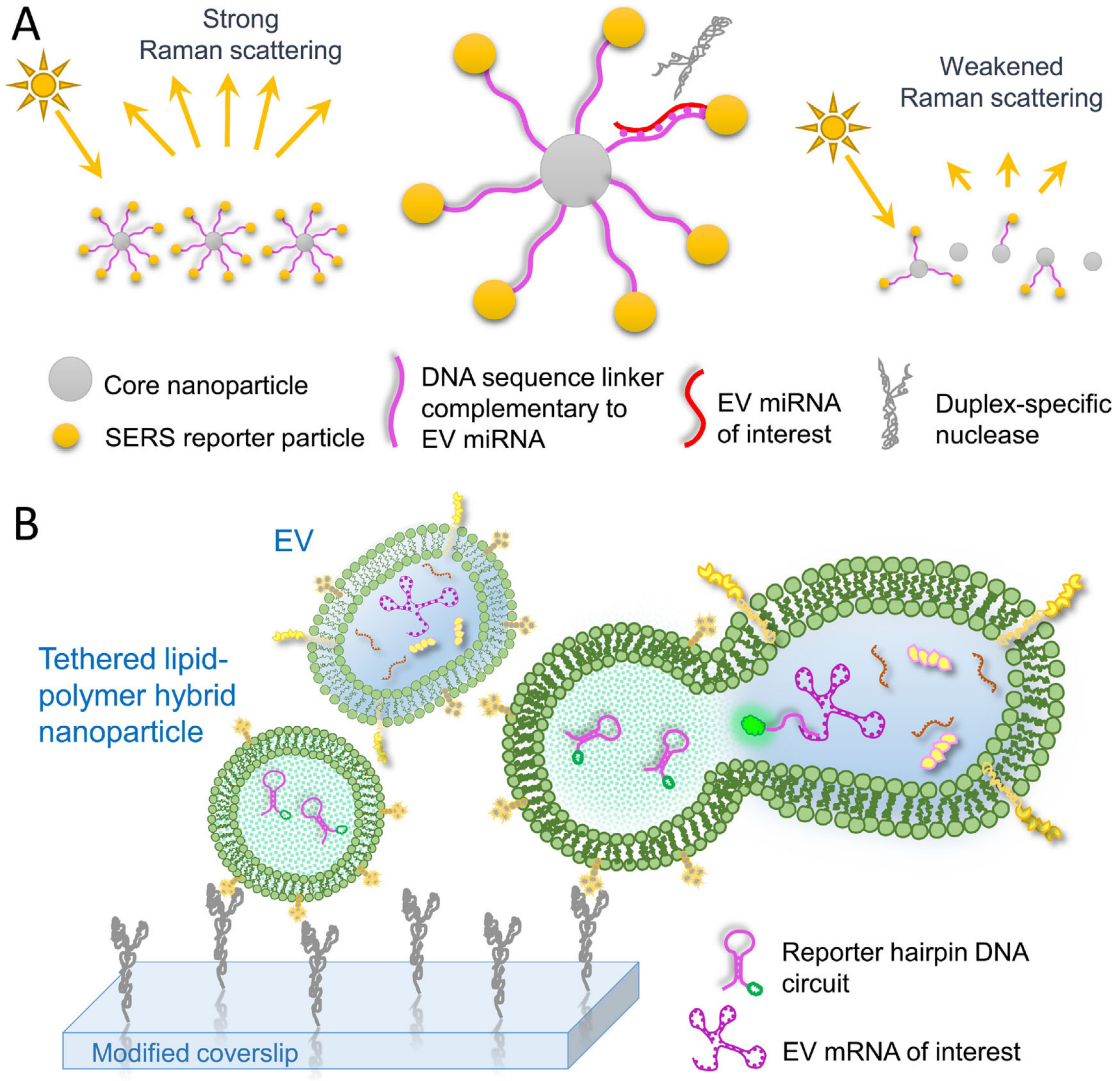
Technique schematics for EV RNA characterization. (A) Schematic of a novel SERS probing system. A core nanoparticle is decorated with SERS reporters using a single‐stranded DNA linker that is complimentary to the target miRNA. Upon presence of EV miRNA of interest, the duplex‐specific nuclease cleaves the hybridized double strands, releasing reporters free from core nanoparticles. The target miRNA quantity is a function of altered SERS signals detected from enriched core nanoparticles. (B) Design of an EV mRNA in situ assay. Immobilized lipid‐polymer hybrid nanoparticles seize and fuse to EVs. Interaction of DNA reporter in hybrid particles with mRNA of interest in EVs produces visible fluorescence for TIRF imaging and EV mRNA analysis

Similarly, SERS is another tool to detect biomarkers with low concentrations, and RNA is one of those that is highly attractive and has been intensely investigated (Abell et al., 2012; Ye et al., 2018). Based on urine‐isolated RNA fragments, Koo et al. established a scoring system for prostate cancer risk prediction using their developed SERS sensor (Koo et al., 2018). Another group employed SERS techniques for miRNA biomarkers associated with primary liver cancer (Zhu et al., 2018). It was not until recently that SERS was applied to analyze EV‐derived RNAs. A head‐flocked gold nanopillar SERS biosensor was reported to detect EV miRNAs closely related to breast cancer. Through introducing locked nucleic acid species as probes, the specificity of the sensing system approached a detection level down to a single‐base mismatch (Figure 6B) (Lee et al., 2019). Although this method had high sensitivity, it required multiple hybridizing and washing steps to construct the “sandwich structure”. To overcome these drawbacks, a few laboratories have developed a simplified strategy for SERS miRNA assays (Ma et al., 2018; Pang et al., 2019). In the probe design, SERS sensitive reporters were conjugated to a core nanoparticle with a single‐stranded DNA linker which was complementary to the target EV miRNA. After incubation with the sample mix, the duplex‐specific nuclease was introduced to specifically cut‐off hybridized DNA strands while releasing the intact miRNA for new cleavage cycling. The cleavage of DNA linker strands separated the SERS reporters from the core nanoparticle, thereafter, reducing signals when measuring recovered core nanoparticles (Figure 7A) (Pang et al., 2019). In such design, the detection of EV miR‐10b levels reached a concentration down to the attomolar (10^–18^; aM) scale and were able to distinguish between pancreatic cancer and healthy individuals (Pang et al., 2019).

#### 2.2.3 Integrated microfluidic chips

One of the many drawbacks associated with the above‐discussed techniques is the requirement for prior (and extra) protocols of EV isolation and RNA extraction. Efforts from laboratories expertized in microfluidic technology have provided some hints to solve these problems. One example applied to EV RNA analysis is represented by the work from Taller and colleagues where they combined on‐chip EV lysis and RNA detection on a single platform for rapid EV‐miRNA analysis (Taller et al., 2015). These investigators applied raw cell culture medium on the chip, where surface acoustic waves then lysed EVs and released miRNA contents. The free miRNAs flowed through to the second chip which captured the target miRNAs using a DNA probe‐functionalized ion‐exchange nanomembrane sensor, which, upon miRNA annealing, would alter the current‐voltage characteristics (Taller et al., 2015). This approach, however, was not able to exclude non‐EV contaminant RNAs, particularly when applied to complex biofluids, rendering uncertainties in further translational and future clinical applications. In a different study, Shao et al. provided a more elaborate configuration for microfluidic‐based EV mRNA analysis (Shao et al., 2015). The integrated platform implemented functions of tumour specific EV enrichment, RNA capture, reverse transcription, and finally a qPCR assay. Notably, with human serum samples directly applied on‐chip from patients with glioblastoma multiforme (GBM), the comprehensive device evidently revealed significant changes in the level of EV‐mRNA transcripts expressing key enzymes involved in DNA repair. This device has provided putative potential for monitoring chemotherapy response for patients bearing GBM (Shao et al., 2015). Taken together, emerging chip‐based EV RNA assays that consolidate multiple functional modules have simplified analytical protocols and fostered the potential translation into diagnostic devices.

#### 2.2.4 Single‐vesicle in situ analysis

Aiming to avoid the multi‐step process of EV isolation and RNA extraction, along with the accompanied RNA contamination and degradation, some groups explored new routes for in situ EV RNA analysis, where the oligonucleotide probe (i.e., molecular beacon) is introduced into single EVs and generates detectable signals from inside intact vesicles for specific RNA quantification. Early attempts delivered specifically designed molecular beacons into EVs via treatment of penetrating reagents, and upon hybridization with the molecular beacon inside the vesicle, fluorescence readout determined the target RNA concentration (Lee et al., 2016; Lee et al., 2015; Zhao et al., 2020). Yet, the sensitivity of detection was limited by the weak signal intensity from individual vesicles and the low resolution of the microplate photometer. He et al. recently advanced this approach by utilizing a DNA catalytic reaction and TIRF microscopy (He et al., 2019). Upon penetration, inactive DNAzymes and fluoro‐quenched substrates were co‐delivered into EVs, where the targeted miRNA initiated the enzymatic reaction and amplified fluorescence signals from single vesicles. TIRF imaging of high signal‐to‐noise ratio enabled single vesicle visualization as well as precise quantification of miRNAs within each single EV (He et al., 2019).

Rather than penetrating the membrane and passively sending the probe into an EV, a more elaborate strategy was achieved by delivery of the probe through fusion of EVs with synthesized probe‐encapsulated nanovesicles. One group designed a lipid‐polymer hybrid nanoparticle with a catalyzed hairpin DNA circuit enclosed. The nanoparticle was tethered on a slide and then captured and fused with EVs through electrostatic interactions. The fusion resulted in a mixing of EV mRNA with the DNA catalyst, which then generated and amplified the fluorescence signals for subsequent TIRF imaging (Figure 7B). The technique was able to detect and quantify glypican‐1 mRNA from serum EVs which classified pancreatic cancer patients from healthy controls (Hu et al., 2017). Inspired by a similar idea, another group engineered a virus‐mimicking nanovesicle with functional proteins embedded on the membrane surface. When incubating with EVs, the engineered vesicle‐associated hemagglutinin‐neuraminidase (HN) recognized and bound sialic acid epitopes on EVs. Then the fusion protein on the engineered vesicles promoted fusion with the EV. When the vesicles fused, the enveloped molecular beacon targeted the specific EV miRNA and fluoresced, consequently elucidating the intensity of the target miRNA within each single EV through flow cytometry (Gao et al., 2019).

#### 2.2.5 Limitations

From these advanced techniques, sensitivity for EV RNA detection has been greatly improved. However, there remain specific concerns. First, the protocol used to enrich or capture EVs from biological samples varies significantly across platforms, leading to a large difference in EV recovery efficiency and enrichment of EV subpopulations. Without carefully designed RNA references and the proper normalization methods, the final EV RNA concentration (or copy number) is not able to be compared across platforms and laboratories. Such variations would render difficulties to establish general EV RNA‐based diagnostic criterion. Second, though above‐discussed techniques for EV RNA detection and quantification are advancing, hunting for new RNA targets as potential biomarkers is still largely relying on omic analysis (e.g., NGS or microarrays), of which the analytical sensitivity has relatively fallen behind that of detection techniques. Such discrepancy between discovery phase and detection techniques needs further attention from EV researchers. Regardless of the challenges discussed, profiling the plethora of EV RNA molecules is still one of the most promising disease biomarkers in the immediate future for clinical translation.

### 2.3 EV protein evaluation

EVs are a major reservoir for circulating proteins in various biofluids. Proteins associated with EVs originate from their parent cells through distinctly regulated processes (Liang et al., 2014). Unlike EV‐RNA, proteins are encapsulated within the EV lumen as well as embedded on the surface of lipid bilayers, which allows for subtyping EVs via their accessible surface markers without undermining their intact structure. Specifically, a few proteins (e.g., CD81, CD63, HSP90, flotillin‐1, and others) have been considered as canonical and universal markers for EVs derived across cell types, while some other proteins (e.g., EGFR, HER2, EphA2, EpCAM, and others) are increasingly exploited to differentiate tumour from non‐tumour EVs. Future research will continue to elucidate tumour‐, tissue‐, and cell‐type‐specific EV markers through various proposed computational approaches leveraging publicly available datasets and then validated experimentally (Ghosh et al., 2017; Zaborowski et al., 2019).

Current tools used to study EV‐proteins include Western blot, enzyme‐linked immunosorbent assays (ELISA), flow cytometry, and mass spectrometry (for EV proteomic profiling). Western blot is widely used as a most reliable method to detect various EV proteins, but the low sensitivity and the necessity for long preparatory phases limits its further clinical application. ELISA is currently clinically used for biomarker research in EVs (Fiandaca et al., 2015) and has improved sensitivity and accuracy for protein quantification (compared to Western blot); however, there is a compromise among sensitivity/specificity, complexity, and the cost. Conventional flow cytometry employs immunolabeled beads to capture EVs and measures EV protein levels through quantifying the scattered and fluorescent light in comparison to a standard. Nanoscale flow cytometry with improved configuration is feasible to assess protein profiles and quantify protein expression level from single EVs. Flow cytometry is clinically practicable, while more efforts are advocated to improve the confidence of small vesicle detection (as discussed above) and to forward the development of EV‐specific controls (Welsh et al., 2020). Mass spectrometry (e.g., liquid chromatography Orbitrap) has been introduced to study EV proteomics, providing insight into the global profiling of EV proteins. However, mass spectrometry may not be immediately suitable for diagnostic or prognostic intentions due to lengthy workflows and lack of established bioinformatic pipelines for quantification. Here, we intend to review some of the more recent methods for EV‐protein analysis, combining novel strategies in the fields of nanomaterials and nanotechnology for possible EV‐biomarker clinical translation. Many of the below‐mentioned studies in this section are summarized in Table 3.

**TABLE 3:** Summary of new techniques for EV protein evaluation

Technique	EV source	EV isolation	EV capture	Probe	Detection instrument required	Ref.
Labelled with Q‐dot‐antibody + fluorescence plate reader	Serum, cell culture	None for serum; UC for cell culture	CD81‐antibody functionalized microplates	Quantum dot‐antibody	Unspecified fluorescence plate reader	(Rodrigues et al., 2019)
Plasmon effect‐induced scattering change from coupled AuNP + dark‐field microscopy	Plasma	None	CD81‐antibody functionalized chip	AuNP‐antibody	Dark‐field microscope, Olympus	(Liang et al., 2017)
Labelled with magnetic NP + microfluidic chip + μNMR	Plasma, cell culture	Differential centrifugation	None	Magnetic NP‐antibody	Lab‐built miniaturized NMR relaxometer	(Shao et al., 2012)
Beads‐captured and labelled + electrochemical sensing by an integrated device	Plasma, cell culture	None for plasma; UC for Cell culture	Antibody functionalized magnetic beads	HRP‐antibody	Fabricated assay device	(Jeong et al., 2016)
Nanostructure‐enhanced EV capture + fluorescence microscopy	Plasma	None	CD81‐antibody functionalized chip	β‐galactosidase‐antibody	3I Spinning Disk Confocal Epifluorescence TIRF inverted microscope, Olympus	(Zhang et al., 2019)
Graphene oxide/aptamer nanoprobes + fluorescence spectra detection	Serum, cell culture	None for serum (filtered with 0.22 μm filter); UC for cell culture	None	Fluorescent aptamer	Fluorescence spectrophotometer, F‐4600, HItachi	(Jin et al., 2018)
Near‐infrared afterglow nanoprobe/quencher‐aptamer complex + detection for long‐lasing emission	Cell culture	UC	None	Quencher‐aptamer	GloMax® Luminometer	(Lyu et al., 2019)
Nanohole array capture + transmission SPR detection	Ascites, cell culture	None for ascites (filtered with 0.2 μm filter); UC for cell culture	CD63‐antibody functionalized chip	None	Customized spectrometer for transmission SPR	(Im et al., 2014)
Transmission SPR	Plasma, cell culture	UC	Antibody functionalized chip	None	Spectrometer, USB4000‐UV‐VIS‐ES, Ocean Optics Inc.	(Yang et al., 2017)
Optical product deposits for signal amplification + transmission SPR	Plasma	None	Aβ42‐antibody functionalized chip	HRP‐CD63 antibody	Spectrometer, Ocean Optics	(Lim et al., 2019)
Fluo‐aptamer label + fluorescence imaging	Serum, cell culture	None for serum; UC for cell culture	Size‐based separation by viscoelastic microfluidics	Fluorescent aptamer	Confocal microscope, UltraVIEW VoX	(Liu et al., 2019)
PEA	Cell culture, seminal plasma, breast milk	UC	None	Oligonucleotide‐conjugated antibodies	Proseek Multiplex, Olink Proteomics	(Larssen et al., 2017)
PEA	Plasma	Acoustic seed trapping	None	Oligonucleotide‐conjugated antibodies	Proseek Multiplex, Olink Proteomics	(Gidlof et al., 2019)
Digital immunoassay	Plasma	Immuno‐capture	None	Antibody‐conjugated microbeads	Simoa HD‐1 analyzer, Quanterix	(Shi et al., 2016)
Digital immunoassay	Cell culture, CSF, plasma	UC, or ExoQuick™ kit, SBI + immune‐capture for plasma	None	Antibody‐conjugated microbeads	Simoa HD‐1 analyzer, Quanterix	(Guix et al., 2018)

Abbreviations: AuNP, gold nanoparticle; CSF, Cerebrospinal Fluid; magnetic NP, magnetic nanoparticle; PEA, proximity extension assays; SPR, surface plasmon resonance; UC, ultracentrifugation.

#### 2.3.1 Antibody‐based assay with enhanced reporting system

A common strategy to quantify EV proteins is to construct a sandwich‐type assay, where EVs are captured by the immobilized antibody against well‐known EV markers such as CD81, CD63 or CD9; then, the captured EVs are incubated with a reporter‐conjugated antibody against the protein of interest; finally, by reading proportional signals, the quantity of EV proteins is determined. Challenges with such assays are consistently associated with scarce amounts of EV proteins of interest and the weakness in multi‐target profiling. Several laboratories have attempted to solve this problem by either a) enhancing the reporter sensitivity or b) utilizing a signal amplification system. For instance, Rodrigues et al. immobilized EVs on a 96‐well plate with CD81 antibody, then detected EV surface proteins with quantum dot‐conjugated antibodies. The quantum dot probes provide a much narrower, yet brighter emission spectra without much concern for photobleaching, allowing detection of trace amounts of multiple tumour‐EV proteins in one run (Rodrigues et al., 2019). Through this technique, the researchers were able to reliably measure EpCAM and EphA2 from EVs and use these markers as classifiers for diagnosing pancreatic cancer from patient serum samples. Similarly, another research group employed HRP‐antibody and electrochemical reduction of 3,3′,5,5′‐tertramethyl benzidine (TMB) as a reporting system in this sandwich‐type assay to convert and amplify the signal (Doldan et al., 2016). Of note, a further enhancement of reporter probes was demonstrated by Liang and colleagues where they immobilized EVs and then labelled EVs with AuNP‐conjugated antibodies. The scattered light from the AuNPs (spheres or rods) was detectable by dark field microscopy, while the co‐labelling of a gold nanosphere and a nanorod on a single EV significantly shifted the wavelength and increased the signal intensity due to the local plasmon effect. This technique allowed dual detection of proteins simultaneously and performed with a LOD as low as 0.2 ng/μl compared to 77 ng/μl with conventional ELISA assays (Liang et al., 2017).

Through a similar principle, Dr. Hakho Lee's Laboratory has advanced the translational potential of EV‐protein quantification through development of an integrated sensor chip for a rapid assay. For monitoring glioblastoma multiforme progression, a compact microfluidic chip was manufactured which was able to process and detect plasma EV proteins via a miniaturized nuclear magnetic resonance (NMR) system. The microfluidic detection system revealed elevated protein expression of EGFR, EGFRvIII, PDPN, and IDH1 R132H on EVs, which discriminated tumour‐derived from control EVs (Shao et al., 2012). Additionally, this group developed a different compact sensor system which was designed for plasma EV screening in the setting of ovarian cancer. The plasma EVs were enriched by CD63 antibody‐coated beads, and then bound with antibodies against target proteins. Upon loading on the sensor device, electrochemical signals with high sensitivity were recorded for protein profiling and measurement (Jeong et al., 2016).

#### 2.3.2 Aptamer‐based EV protein probes

**FIGURE 8:**
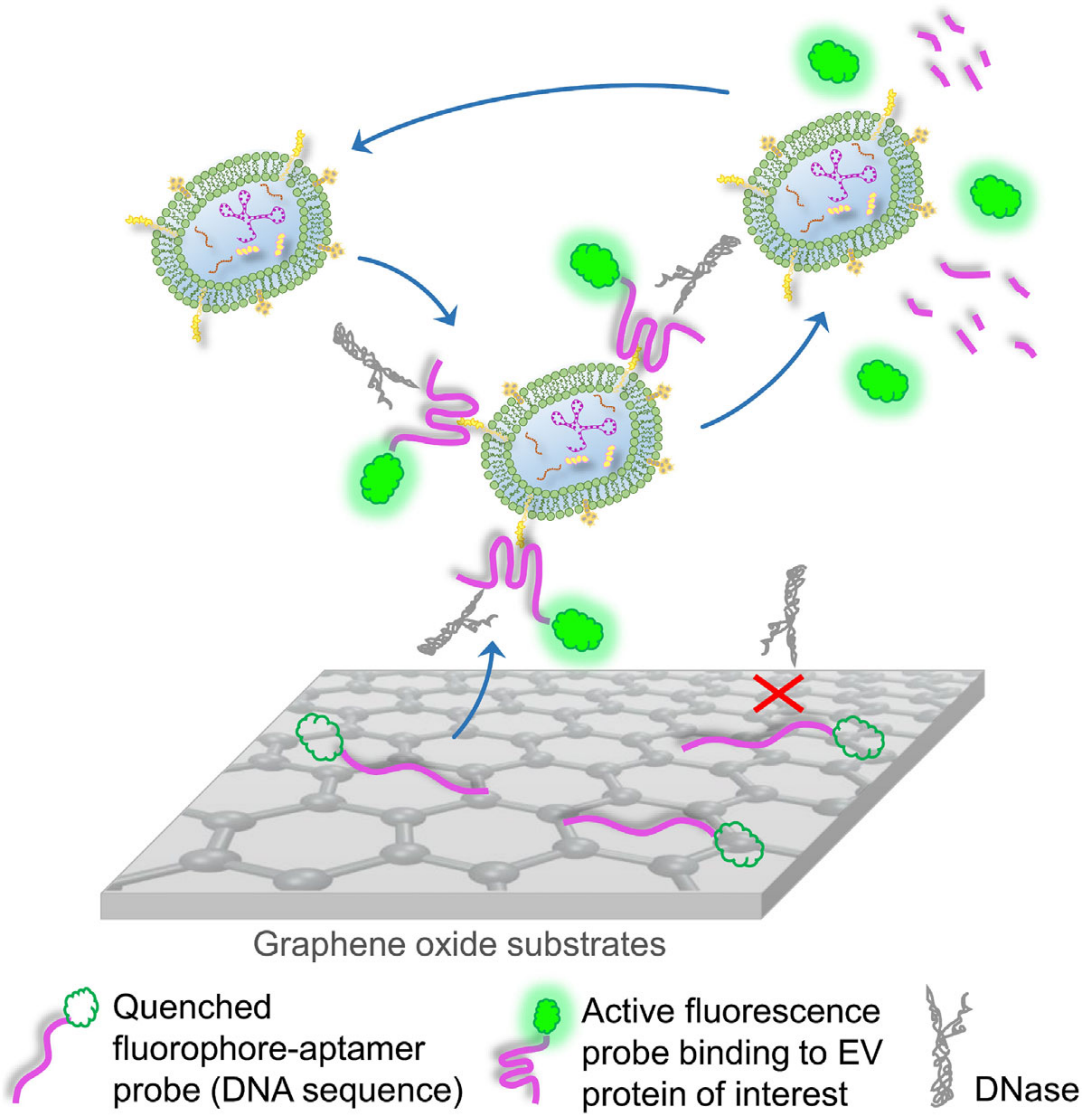
An EV protein assay based on aptamer‐affinity. Aptamer‐attached dyes are quenched on graphene substrates. Upon presence of EVs, dye aptamers detach from substrates and bind to EV proteins of interest. DNase then cleaves the EV‐bound aptamer, releasing the dye molecule and allowing the EV to attract more aptamers from the substrate. Measurement of free dyes reveals the quantity of target EV proteins

**FIGURE 9:**
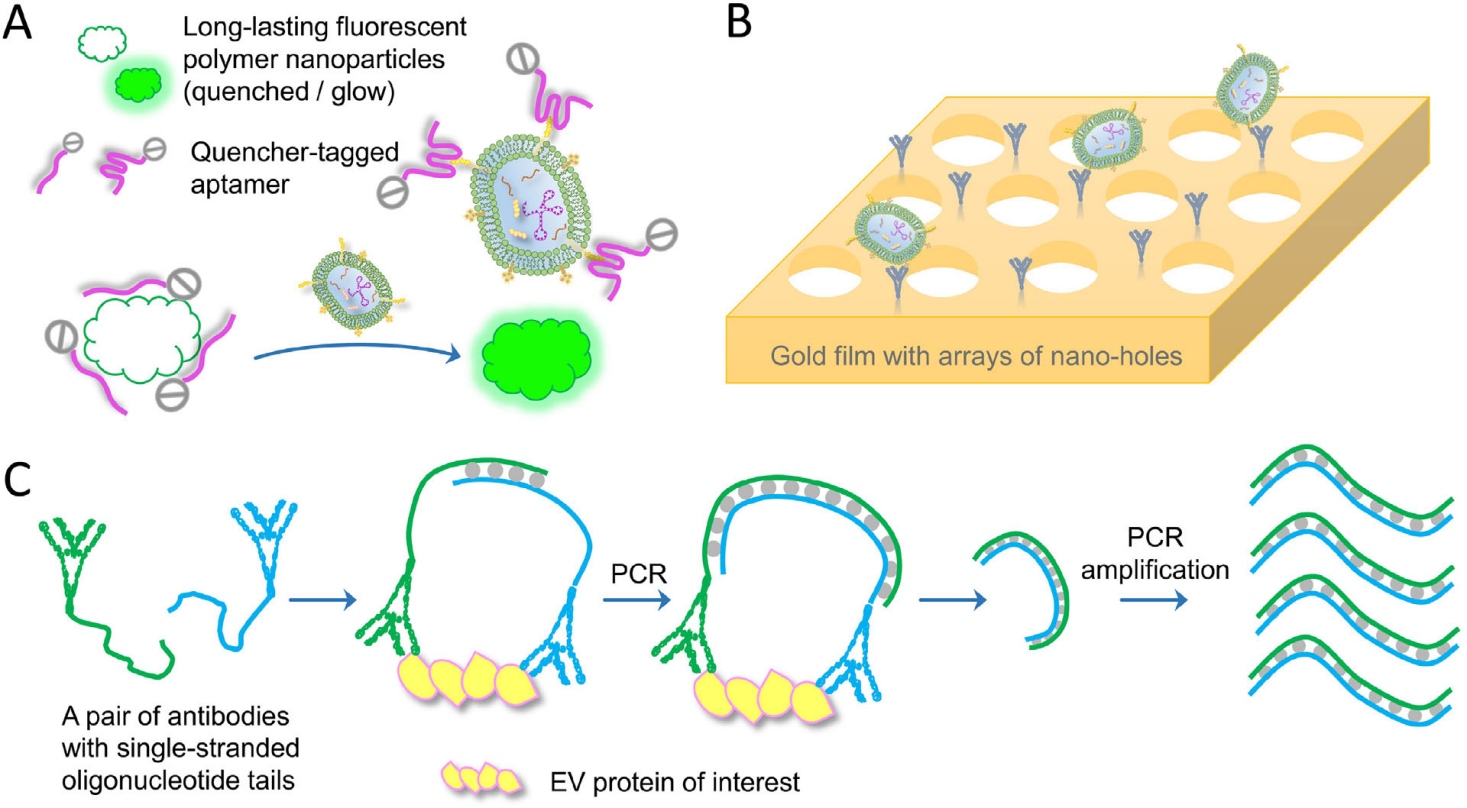
Schematics of assorted techniques for EV protein assessment. (A) A design of aptamer‐based EV protein assay. The quencher‐tagged aptamers initially associate with a fluorescent core particle. By loading an EV sample, aptamers detach from the core particle and retrieve the fluorescence signals, which indicates the quantity of the target EV protein. (B) Nanohole array‐assisted SPR nano‐biosensor for EV protein quantification. A metal film that has arrays of nano‐sized holes with diameters similar to EVs’ is used to capture EVs through the antibody affinity to the EV protein of interest. EVs’ attaching on the film surface will alter the SPR intensity, demonstrating the target EV protein amounts. (C) Assay workflow of the PEA technique for protein quantification. The target protein is bound with a pair of antibodies, on which the attached oligonucleotides anneal and extend, forming a double‐stranded DNA sequence. The copy number of this unique sequence represents the quantity of the target protein. Upon qPCR amplification of sequences, the quantification of target proteins is achieved

While protein marker detection largely relies on target‐specific immunoaffinity of assorted antibodies, aptamers are another protein‐binding motif which have garnered increased attention in recent years. Because of their nucleotide‐based structure, aptamers are more conformationally stable, and more accessible to various chemical modifications as compared to most antibodies. Thus, several groups have developed EV‐protein assays with high sensitivity utilizing target‐specific aptamers. Interestingly, Jin et al. designed a sensor chip coated with graphene oxide substrates that could absorb fluorophore‐aptamer probes (Figure 8). Binding of the aptamer probes with graphene oxide quenched the fluorescence. In the presence of EVs, aptamer probes detached from the graphene substrate, bound EV proteins and fluoresced. Then, the DNase that co‐existed in the reaction mixture digested EV‐bound aptamers, but not graphene oxide‐confined ones, and released the fluorophores. The EVs were then able to attract additional probes to amplify the fluorescence signals. With different aptamers, the sensor was able to profile the EV surface protein patterns with high sensitivity (Jin et al., 2018). Another example was reported by Lyu et al. where long‐lasting fluorescent polymer nanoparticles were synthesized to hold quencher‐tagged aptamers via electrostatic interactions. Upon addition of EVs, quencher‐aptamers discharged from the polymer and activated fluorescence (Figure 9A) (Lyu et al., 2019). Aptamer‐based EV‐protein evaluation is attractive in multiple settings, however, it is primarily limited by the availability of published and confirmed aptamer sequences specific to EV proteins. Otherwise it could be a time‐consuming procedure to screen for and determine new sequences. Regardless, employment of aptamer probes for translational applications of profiling EV‐proteins is still believed to hold a promising future.

#### 2.3.3 SPR in application of EV protein detection

Likewise, the SPR technique was demonstrated in the application of detecting EV proteins. A chip design was reported that utilized an ultrathin gold film patterned with arrays of periodic nano‐sized holes (Figure 9B). The thickness of the metal film ranged in several hundred nanometers and the diameter of the nanoholes were approximately 200 nm. The surface of the metal film was functionalized with antibodies that captured and enriched EVs. Surface binding of the target EVs changed the local refractive index, thereby affecting plasmon resonances of the sensor chip. By measuring the wavelength and intensity of transmission light through the film, the amount of EV proteins attached could be deduced from standard calibration curves (Im et al., 2014; Yang et al., 2017). Dr. Huilin Shao's laboratory further advanced this technology and had exhibited intriguing results in correlating circulating EV‐amyloid β (Aβ) levels with a PET imaging‐based diagnosis for Alzheimer's disease patients. Similarly, using a nanohole‐arrayed metal film as the substrate for a sensor chip, the researchers captured both EV‐bound and free Aβ via an immobilized anti‐Aβ antibody. Further, Aβ‐associated EVs were labelled with HRP‐tagged CD63 antibodies, which then catalyzed the enzymatic reaction and produced a plethora of optically detectable deposits locally around EVs, greatly enhancing the SPR signals. With raw plasma applied on‐chip, researchers found that EV‐associated Aβ, but not unbound or total circulating Aβ, manifested a good prognosis of Alzheimer's disease and other cognitive impairments (Lim et al., 2019).

#### 2.3.4 EV protein profiling with proximity extension assays

One prominent challenge that has impeded the advancement of EV protein profiling is associated with the modest number concentration of EVs available for testing and the limited amount of proteins within single vesicles. The proximity extension assay (PEA) was proposed a few years ago which notably improved the sensitivity of protein detection by means of converting the protein measurement into a DNA sequence quantification (Figure 9C) (Assarsson et al., 2014; Lundberg et al., 2011). In this assay, a pair of antibodies against the target protein, ideally two monoclonal antibodies binding distinct epitopes, were individually labelled on Fc ends with a single‐stranded oligonucleotide, and parts of the free end regions complementary to each other. Upon incubation with samples, the target protein would be recognized by the antibody pair. Due to the oligonucleotide proximity, the free end regions hybridized and extended via a polymerization reaction. The newly formed double‐stranded DNA fragments carry unique barcode for each target protein, allowing for plenty of target proteins to be analyzed simultaneously (up to ≥ 90 proteins in one panel). Finally the barcode sequences were amplified by qPCR, and the readout was proportionally correlated to the protein of interest for quantification (Assarsson et al., 2014; Lundberg et al., 2011).

The PEA technique has been employed in EV protein evaluations. A study using PEA characterized EV protein patterns from multiple cell lines, and found different EV protein profiles across different EV sources, with bioinformatics informing cellular origin of profiled EVs (Larssen et al., 2017). A few newly published articles have studied blood plasma or serum EV proteomics via PEA in the disease settings of neurocognitive disorders (Sun et al., 2019), glioma (Chandran et al., 2019), myocardial infarction (Gidlof et al., 2019), and ovarian cancer (Dorayappan et al., 2019) where panels of EV‐protein biomarkers that distinguish between different clinical populations have been identified. Such frequent reports demonstrate the value of PEA in EV protein analysis, however, this technique requires sophisticated design and synthesis of oligonucleotide‐tagged antibody pairs, as well as multiple experimental steps, thus currently only available from commercial vendor services. In addition, the protein panels to be detected are predefined without optimization for EV‐protein features, further limiting its efficient practice in the EV field.

#### 2.3.5 Digital immunoassay for absolute protein quantification

Inspired by the similar principle applied for digital PCR, a single protein molecule immunoassay was developed featuring the absolute quantification. Such approach, which is commercially available, employed antibody‐conjugated microbeads as the medium to sequester protein molecules (Rissin et al., 2011; Rissin et al., 2010). The number of microbeads far exceeds the number of target proteins, enabling a small percentage of beads to be bound with one target molecule, while many beads have no target bound. All the beads are then loaded into arrays of fL‐sized wells which hold no more than one bead per well. With the substrate added, only the beads with protein complexes attached produce signals, yet the rest of the wells are silent. By counting the positive and negative wells, the absolute quantification of target protein is achieved (Yelleswarapu et al., 2019). This technique has assisted discovery and assessment of EV proteins as critical disease biomarkers. Shi and colleagues isolated L1CAM‐positive EVs from the plasma, which were considered to be putatively derived from central nervous system components. By utilizing the digital protein analysis they observed higher expression of tau in EVs collected from Parkinson's disease patients. Interestingly the EV‐tau value correlated well with CSF‐tau expression (Shi et al., 2016). A different laboratory used the same technique and thoroughly characterized full‐length and truncated EV‐derived tau, along with free‐floating tau species from plasma and CSF samples (Guix et al., 2018). The capability of femtomolar‐level detection and absolute measurement allows the digital protein immunoassay to provide more robust insights into potential EV‐based protein biomarkers for a variety of acute and chronic diseases.

#### 2.3.6 Limitations

From the perspective of clinical translation, PEA and more‐widely‐used mass spectrometry methods are helpful in early protein biomarker discovery, while protein sensors would be good for multiplex panel detection as diagnostic kits. However, either of these applications needs to be adopted with careful consideration of the method and efficiency of EV isolation. Different subpopulations of EVs in biofluids may give distinct protein expression patterns and profiling, thus present various challenges for specificity concerns. Overall, EV‐protein assays for diagnostic purposes are encouraging and present one of the most opportune systems for future clinical translation, yet much technological improvements integrating consistency of EV subtyping and enrichment are needed before their widely clinical acceptance.

### 2.4 EV lipids as disease biomarkers

Beyond proteins and nucleic acid species, the lipid components that construct the EV bilayer membrane are another essential element of EV, and could be profiled for disease detection. The metabolic pathways that regulate lipid biosynthesis and transport are closely related to cell physiological status (Fernandez‐Murray & McMaster, 2016), and EV lipids have apparent variations in different pathophysiological states of parent cells or tissues. For example, the abnormal deficiency of phosphatidylinositol‐3‐phosphate, a phospholipid that regulates lysosomal and autophagic functions in neuron, represents a pathological feature of neurodegenerative disorders, and EVs from these dysregulated neurons display an altered lipidomic profile (Miranda et al., 2018). Of note, accumulating studies have demonstrated that instead of simple replica of parent cellular membrane, EV lipids have preferentially enriched classes and species compared to parent cells (Haraszti et al., 2016), which add another layer of complexity in EV‐lipid biology and pathology.

However, identifying reliable EV‐lipid biomarkers is not trivial, and such challenges arise from several aspects. First, similarly to protein and RNA analysis, the EV population purified from biofluids is often heterogeneous. Co‐isolation of lipid droplets or mitochondria may introduce high levels of cholesteryl ester, triacylglycerol, and cardiolipin (Daum, 1985; Horvath & Daum, 2013), while for even more complex biofluids, such as blood, isolated EVs have greater opportunity for high lipoprotein contamination, which have similar size (i.e., VLDL or LDL), or density (i.e., HDL) compared to EVs (Onodi et al., 2018; Sodar et al., 2016). Inclusion of lipoproteins in the final EV isolate results in plenty of non‐EV phospholipids and sphingomyelin, and therefore reduces the selectivity and specificity of detection. The second issue arises from the limited tools for lipid identification and quantification. Lipids have high diversity in terms of chemical structure and compositional ratio. Major membrane lipids include glycerophospholipids, sphingolipids and sterols (mainly cholesterols), and each class of the first two further have variation in head groups, sphingoid bases, fatty acid chain length, double bond number and position, and addition of oligosacchrides found in glycosphingolipids (Harayama & Riezman, 2018). The complexity of lipid diversity leaves mass spectrometry as nearly the only efficient methodology to study EV lipidomics. In addition, the third aspect arises from the asymmetric distribution of lipids in the two leaflets of the EV membrane. Although distinct lipid compositions in plasma membrane leaflets have been well documented, to our best knowledge, very little is known about that of EVs (Skotland et al., 2017).

To date, the most common workflow for EV lipidomics involves prior lipid extraction via organic reagents, separation via liquid chromatography, and the final detection via mass spectrometry. Early studies mostly focused on in vitro EV samples derived from various cell lines, and by comparison several lipid classes are presumably conserved in EVs. These lipids include cholesterol, sphingomyelin, ceramide, and phosphatidylserine, while others like phosphatidylcholine tend to be excluded from EVs (Brouwers et al., 2013; Haraszti et al., 2016; Llorente et al., 2013; Lydic et al., 2015; Osteikoetxea et al., 2015; Trajkovic et al., 2008). Recently, EV lipid investigations in clinical settings have uncovered their potential as clinical disease biomarkers. Hough et al. analyzed EV lipidomics from asthmatic bronchoalveolar lavage fluid specimens and found ceramides and glycerophospholipids to be downregulated in the disease condition (Hough et al., 2018). Similarly, another study utilized liquid chromatography‐mass spectrometry (LC‐MS) for urinary EV lipids collected from prostate cancer patients, and found upregulation of phosphatidylserine and lactosylceramide in patients compared to healthy controls (Skotland et al., 2017). Besides LC‐MS, other techniques for detecting EV lipids have been scarcely reported. Singhto and colleagues attempted to separate EV lipid species through thin layer liquid chromatography, and then recovered each as single bands for matrix‐assisted laser desorption ionization time‐of‐flight mass spectrometry (MALDI‐TOF MS) analysis, and the result was validated by dot blot with antibodies against specific lipid classes (Singhto et al., 2019). The problem confining such lipid immunoassays partly results from limited antibody specificity and low binding affinity. Sharma et al. developed a recombinant dimerized antibody that had IgG Fc domains fused with the phosphatidylserine recognition domain β2GP1, which significantly enhanced the binding affinity to EV‐associated phosphatidylserine (Sharma et al., 2017). Taken together, though LC‐MS‐independent techniques have emerged to explore EV lipid biomarkers, there still exist many challenges before employing them as alternatives to LC‐MS.

On the other hand, even for the widely used mass spectrometry platform, several experiment details and criteria need to be seriously considered. To avoid mistakes in lipid annotation, high‐resolution mass spectrometry is encouraged. Currently tandem mass spectrometry (involving triple quadrupole or quadrupole‐TOF) for EV lipidomic research is becoming the minimum instrumental requirement to ensure the reliable assignment of chemical structures and functional groups. After pilot experiments with untargeted discovery, carefully designed studies with targeted validation in larger cohorts are crucial. Additionally, for quantitative purposes, stable isotope‐labelled internal standards of each lipid class, or species, are necessary to correct for the efficiency during lipid extraction, absorption, ionization, and other processes. Although it is ideal to use standards for each lipid structure of interest, most of the reported studies only used structural analogs representing a given lipid class for semi‐quantitative analysis. Overall, EV lipid‐based biomarkers, either in the discovery phase or detection platforms, remain largely unexplored, yet hold promise as future biomarkers in clinical practice.

### 2.5 Whole EV fingerprint profiling

Some researchers have made a distinct approach to advance EV‐based biomarker detection through depicting the whole EV spectroscopic profile instead of dissecting into specific cargo molecules. Under certain detection methods, the entire chemical components of a single or subgroup of EV(s) collectively present a unique spectroscopic pattern to identify the pathologic origin, to which we refer as a “fingerprint” in the following description. Raman spectrum was reported as a detection tool to portray the molecular signature of single EVs (Gualerzi et al., 2019; Gualerzi et al., 2017). In a reported assay jointly utilizing the technique of optical tweezers, EVs from an unpurified sample were stably trapped in the center of a focused laser beam with three single vesicles at most. The number of trapped single vesicles was detected by Rayleigh scattering light, and the molecular feature was snapped by Raman spectroscopy. The peak shift pattern reflected the fingerprint of specific EV subclasses which was able to present distinguishable characteristics between tumour‐derived EVs, red blood cell‐derived EVs, and lipoproteins (Enciso‐Martinez et al., 2020; Enciso‐Martinez et al., 2020).

Similarly, infrared spectroscopy (IR) was reported to characterize EV profiles. Kim and colleagues employed atomic force microscope infrared spectroscopy (AFM‐IR) to probe the compositional signature of a single EV and certain EV subgroups (Kim et al., 2019). This technology measured the IR absorption spectra with nano‐scale resolution from several distinct points on a single EV, and also generated an AFM height image featuring the topographical landscape of a single EV. The peaks from each single EV's IR spectrum were correlated with different classes of EV molecules ranging from RNAs to proteins to lipids, forming the unique fingerprint of particular EV subpopulations (Kim et al., 2019). Another study by Paolini et al. examined EVs derived from two cell lines using Fourier‐transform Infrared spectroscopy (FT‐IR), and principal component analysis of EV's FT‐IR spectrum identified EV subgroups corresponding to their sizes and cells of origin (Paolini et al., 2020). These approaches are thought to potentially identify EV subpopulations and detect subtle differences in disease conditions through monitoring changes in whole vesicle IR spectra absorbance patterns. However, massive curation of “fingerprint” baselines needs to be annotated in a database to serve as validated hallmarks (i.e., biomarker signatures) for particular diseases. Interestingly, imaging analysis of EV spectra through machine learning has emerged (Shin et al., 2020) and may provide novel insights into disease progression in a more facile manner.

Besides Raman‐ and IR‐based EV fingerprinting, a similar strategy utilizing MALDI‐TOF MS to study intact EV features was reported by Zhu and colleagues (Zhu et al., 2019). MALDI is a mild ionization technique which generates minimum fragments of analytes, therefore, allowing identification of large biomolecules, such as proteins through peptide mass fingerprinting (Webster & Oxley, 2012; Zenobi & Knochenmuss, 1998). However, conventional protein analysis by MALDI‐TOF MS requires protein separation by 2‐D gel electrophoresis, which increases the complexity of analysis. Researchers applied intact EVs on MALDI‐TOF MS without lysis and cargo extraction, producing a collection of highly mixed ion peaks that represented all EV‐components detected. To better interpret the MALDI‐fingerprint, researchers did top‐down and bottom‐up proteomic MS in parallel and managed to assign specific proteins to featured ion peaks. Interestingly, when comparing EV MALDI‐fingerprints between melanoma patients (*n* = 3) and healthy controls (*n* = 3), 11 peaks were recurrently overexpressed in tumour subjects (Zhu et al., 2019). This technique for EV fingerprinting is superior in terms of short processing time and high throughput, but similar to the above‐discussed IR‐spectrum platform, extensive accumulation to unravel the complexity in clinical samples needs to be fulfilled before constructing a reliable diagnostic criterion.

## 3 REFERENCING SYSTEM FOR EV ANALYSIS

As described throughout this review, quantification is a key element for translating EV‐based biomarkers into clinical practice and applicability. Although the above‐reviewed techniques progress towards detection sensitivities at the pico‐ or even femto‐scale, the normalization methods significantly vary from laboratory to laboratory. Normalizing the quantity of EV‐RNAs/proteins/lipids detected of interest to the original sample volume (i.e., ml of biofluid), original EV number concentration, or the total amount of original EV‐RNAs/proteins/lipids, can be a valuable index for potential diagnosis. However, there exist lack of standardized reporting of the efficiency for EV recovery and molecular targets extraction. For instance, human blood plasma contains an upwards of 10^9^ to 10^10^ vesicles per milliliter, with most used isolation methods achieving a recovery efficiency lower than 10%, which has further discrepancy among methods, batches, and operators (Arraud et al., 2014; Berckmans et al., 2019; Holcar et al., 2020; Jamaly et al., 2018). Without elucidating the efficiency of EV recovery and target extraction, the resulting quantification data will not be cross‐referenced among laboratories and then has limited biological and clinical significance.

Establishing a referencing standard for EV/EV‐molecule quantification is critical to the field (Valkonen et al., 2017). One straightforward solution is to incorporate “spike‐in” reference vesicles before processing EV enrichment and analysis. Early attempts employed the use of polymer beads or liposomes with similar size of EVs, whereas the density, refractive index, and biological properties were difficult to meet the needs (Gardiner et al., 2013; van der Pol et al., 2014; Varga et al., 2018). Recently, a group developed a niosome‐cored EV mimetic with surface decorating CD81 or CD63 extracellular loop domains (Lozano‐Andres et al., 2019). The enhanced synthetic vesicle reference presented similar properties in immunoassays, flow cytometry or NTA, while the spike‐in assessments in real samples were not demonstrated. Dr. An Hendrix's laboratory proposed the production of a recombinant vesicle derived from gag‐EGFP‐transfected HEK293T cells which, once spiked in plasma or urine samples, were trackable through fluorescence NTA (against EGFP), immunoassay (against surface proteins), and qPCR (against EV internal EGFP mRNA) (Geeurickx et al., 2019). With recombinant EVs spiked in, the efficiency of EV isolation and the RNA/protein/lipid extraction has been elucidated and incorporated into the calculation and normalization, providing more accurate quantifications. Nonetheless, compared to artificial reference materials, engineered cell‐derived reference EVs remain to be inferior in terms of cell culture cost, inter‐batch consistency, and scale‐up production, hampering its broad application. At the moment when “reference materials” were applied within limited and discrete laboratories, “vesicles test samples” may be a better term describing its current commission from a metrological viewpoint. Eventually, establishment of EV reference system in the field requires the calibration of arbitrary units from individual instruments and laboratories to the International System of Units (SI), which can only be achieved using traceable reference materials with well‐characterized properties and a known uncertainty. Production of traceable reference materials involves specialistic manufacturers and a close collaboration with metrology institutes where SI unites are defined. Expressing EV data that have a known uncertainty with assistance of reference materials would be a leap forward to the field, and of great importance when considering EV/EV‐molecule quantification for applications of clinic diagnostic purposes (Welsh et al., 2020).

## 4 DISCUSSION AND FUTURE DIRECTIONS

The growing understanding and application of EV welcome a broad range of novel technologies and multi‐disciplinary investigators. Development of various nanobiosensors, signal amplification strategies, and sensitive probe designs have markedly enhanced the LOD for measuring the EV number concentration as well as profiling the cargo molecules. However, despite the advancements of these specific EV assessment methods, translation of EV assays for clinical diagnosis requires further considerations (Ayers et al., 2019; Yekula et al., 2020). First, standardization needs to be established from the pre‐analytical processing steps including whole blood (or other biofuild) handling, interference of hemolysis, or inspection of platelet contaminations. Second, and more importantly, EV isolation and enrichment procedures are encouraged to be detailed and specified. Efforts from the EV‐TRACK Consortium established the knowledgebase and stressed the significance of transparently reporting the EV methodology (Consortium et al., 2017), and guidelines for Minimal Information for Studies of EVs (MISEV) have reached a broad consensus in the field for standardization (Lotvall et al., 2014; Thery et al., 2018; Witwer et al., 2017). Further, the development of novel methods for EV isolation and enrichment is of great demand to uncover EV subgroups, small EVs, or even non‐membranous secreted entities (e.g., exomeres), which provides a purer set of analytes for detection and will finally benefit precision diagnostics. Lastly, the reproducibility of an EV‐detection technique needs to be comprehensively demonstrated. It should comprise essential performance characteristics according to International Organization for Standardization (ISO) standard 15189, which is a widely accepted regulatory requirement for medical laboratories (Antonelli et al., 2017; UKAS 2018). Additionally, the technique or assay should be tested for specificity and sensitivity in a large and diversified cohort instead of limited proof‐of‐concept sample populations. Despite these challenges, EV‐based biomarker assays for clinical utility are progressing toward clinical development and implementation. One example is demonstrated through the receipt of a Breakthrough Device Designation from the United States Food and Drug Administration for the first EV‐based liquid biopsy of prostate cancer from Exosome Diagnostics, Inc. (ExoDx Prostate *IntelliScore*) (Tutrone et al., 2020). Collectively, through multi‐disciplinary collaborations in cell and molecular biology, engineering, and medicine, we expect a promising future for clinical translation of EV‐based biomarkers for liquid biopsy diagnosis.

## CONFLICTS OF INTEREST

The authors report no conflict of interest.

## AUTHOR CONTRIBUTIONS

Yaxuan Liang and Brandon M. Lehrich collaboratively wrote the article and contributed equally to this work.
